# Synthesis and Biological Properties of Ferrocenyl and Organic Methotrexate Derivatives

**DOI:** 10.1021/acsomega.4c03602

**Published:** 2024-07-23

**Authors:** Karolina Rózga, Andrzej Błauż, Daniel Moscoh Ayine-Tora, Ernest Puścion, Christian G. Hartinger, Damian Plażuk, Błażej Rychlik

**Affiliations:** † Department of Organic Chemistry, Faculty of Chemistry, University of Lodz, 12 Tamka, 91-403 Łódź, Poland; ‡ Cytometry Lab, Department of Oncobiology and Epigenetics, Faculty of Biology and Environmental Protection, University of Lodz, 141/143 Pomorska, 90-236 Łódź, Poland; § School of Chemical Sciences, University of Auckland, Private Bag 92019, Auckland 1142, New Zealand; ∥ Department of Chemistry, University of Ghana, LG 56 Legon-Accra, Ghana

## Abstract

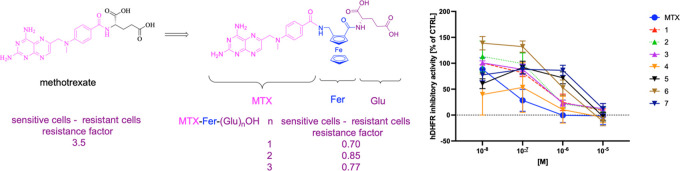

Synthesis and biological activity of two series of modified side chain methotrexate (MTX) derivatives are presented, one with a ferrocenyl moiety inserted between the pteroyl and glutamate portions of the molecule and the other with glutamate substituted for short chain amino acids. Ferrocenyl derivatives of MTX turned out to be rather moderate inhibitors of dihydrofolate reductase (DHFR) although molecular modeling suggested more effective interactions between these compounds and the target enzyme. More interestingly, ferrocene-decorated MTX derivatives were able to impede the proliferation of four murine and human cell lines as well as their methotrexate-resistant counterparts, overcoming the multidrug resistance (MDR) barrier. They were also able to directly interact with Abcc1, an MDR protein. Of the amino acid pteroyl conjugates, the γ-aminobutyric acid derivative was an efficient inhibitor of DHFR but had no effect on cell proliferation in the concentration range studied while a taurine conjugate was a poor DHFR inhibitor but able to affect cell viability. We postulate that modification of the methotrexate side chain may be an efficient strategy to overcome efflux-dependent methotrexate resistance.

## Introduction

The era of cancer chemotherapy started in 1947 when Sidney Farber purposely used synthetic folic acid analogues aminopterin and methotrexate (MTX, amethopterin) for the treatment of acute leukemia in children.^1^ The latter compound is still widely used in medicine. It is currently indicated not only for the treatment of acute lymphoblastic leukemia or other types of cancer but it is also used in autoimmune and inflammatory diseases such as rheumatoid arthritis and psoriasis or for the management of ectopic pregnancy.^2^ Methotrexate binds to and practically irreversibly inhibits dihydrofolate reductase (DHFR), a key enzyme in folic acid metabolism reducing dihydrofolate to tetrahydrofolate at the expense of β-NADPH,^3^ thus impeding DNA replication by decreasing the available nitrogen base pool. Due to high structural similarity to folic acid, MTX undergoes poly(glutamylation)^4,5^ catalyzed by folylpolyglutamate synthetase (FPGS), a process that originally evolved to retain folic acid within the cell. Poly(glutamylated) forms of methotrexate are recognized by and also inhibit other folate-dependent enzymes, i.e., thymidylate synthase^6^ and phosphoribosylaminoimidazolecarboxamide formyltransferase,^7^ thus increasing the pharmacodynamic potency of the drug. The clinical usefulness of MTX is, however, limited not only by its high systemic toxicity but, more importantly, by the reduced sensitivity of target cells toward this antifolate. The resistance to methotrexate may originate from different processes, including amplification and elevated *DHFR* gene expression,^8^ reduced MTX uptake rate,^9^ impaired poly(glutamylation),^10^ resulting from genetic alterations of *FPGS*,^11^ and augmented efflux of MTX and/or its active metabolites.^12^ The last of these mechanisms is of special clinical importance as it is usually mediated by low-specific membrane transporters of the ATP-binding cassette (ABC) superfamily and may lead to multidrug resistance (MDR), i.e., decreased susceptibility of a target cell to a number of structurally different compounds of variable modes of action. It was demonstrated that MTX and its oligo(glutamylated) forms are recognized and actively exported outside the cell by ABCC1 and ABCC3,^13^ ABCC4^14^ (MTX and MTX-Glu but not MTX-Glu_2_) and ABCC5^15^ (MTX, MTX-Glu, and MTX-Glu_2_ but not MTX-Glu_3_) as well as wild type and some variants of ABCG2^16−18^ (MTX, MTX-Glu_1–3_). The practical importance of MDR in cancer therapy can be exemplified by the study of Jaramillo et al. demonstrating that elevated expression of ABCC4 and ABCG2 negatively impacted the MTX response in pediatric patients with acute lymphoblastic leukemia.^19^

**Figure 1:**
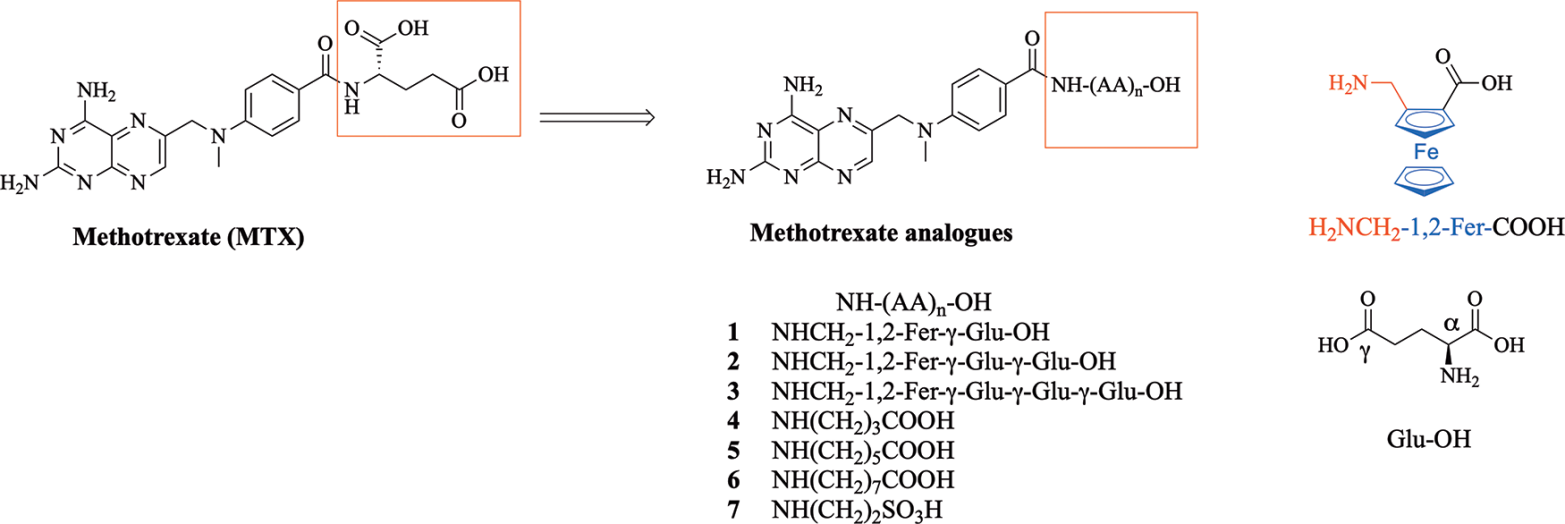
Methotrexate (MTX) and its derivatives **1**–**7** studied herein. AA = amino acid.

Although modern cancer treatment is focused more on targeted therapies and less on chemotherapy, the widespread use of methotrexate as an immunomodulatory compound prompted us to seek MTX derivatives that can overcome multidrug resistance. We wanted to preserve the active pteroyl fragment of the molecule and concentrate on the glutamate side chain as a factor important for the recognition of MTX and its glutamylated forms by MDR transporters. An appealing possibility was to introduce a metallocenyl moiety into a methotrexate molecule. Such a strategy turned out to be fruitful in the case of modification of many natural products (for a review, see e.g., ref (20)) and—except for a single study^21^—was not employed for folates so far. We demonstrated previously that inserting an organometallic component in a biologically active molecule may dramatically alter its properties, e.g., turn a vitamin into a toxin^22^ or change the molecular target of the parental compound.^23^ Therefore, we investigated if the introduction of a ferrocenyl moiety affected the biological properties of MTX (Figure 1, compounds **1**–**3**). We also substituted the glutamate moiety in methotrexate for another unbranched amino acid (Figure 1, compounds **4**–**7**) as this kind of modification was also not widely studied despite extensive work done in this field by Rosowsky and colleagues in the 1980s and 1990s.^24−26^

## Results and Discussion

### Synthesis

**Scheme 1:**
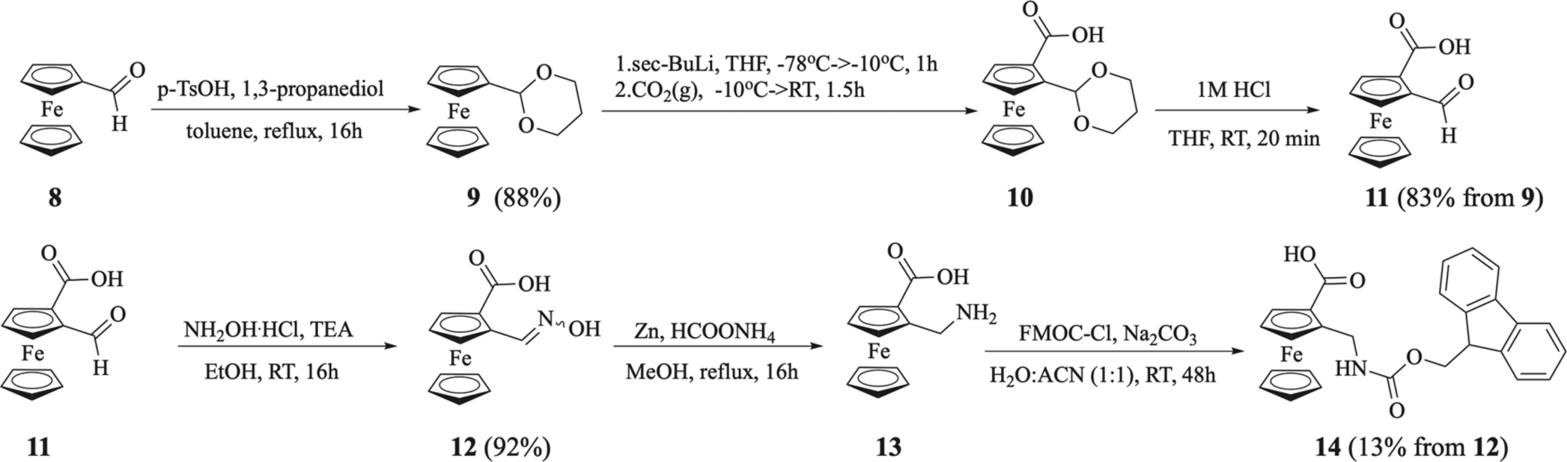
Synthesis of *rac*-*N*-Fmoc-2-Aminomethylferrocene-1-carboxylic Acid **14**

The ferrocenyl conjugates **1**–**3** were synthesized from *rac*-*N*-Fmoc-2-aminomethyl-1-ferrocenecarboxylic acid **14**. The latter compound was prepared in multistep reactions according to Scheme 1. First, ferrocenecarboxaldehyde **8** reacted with 1,3-propanediol and TsOH produced acetal **9** in 88% yield.^27^ The latter compound was lithiated with *sec*-BuLi, followed by gaseous carbon dioxide to afford acid *rac*-**10**. Then, the acetal moiety of crude **10** was removed by acidic hydrolysis with 1 M hydrochloric acid in THF, affording *rac*-2-formyl-1-ferrocenecarboxylic acid **11** in 83% overall yield. The further reaction of **11** with an excess of hydroxylamine hydrochloride and triethylamine afforded oxime **12** in 92% yield (using sodium hydroxide instead of triethylamine gave **12** in only 54% yield), isolated as a mixture of the *anti*- and *syn*-isomers (87:13 based on the ^1^H NMR spectra of crude product). The formation of oxime **12** was confirmed by ^1^H, ^13^C{^1^H} and ^15^N NMR spectroscopy. In the ^1^H–^15^N HMBC spectrum of pure **12**, isolated as the *anti*-isomer, we observed a ^15^N signal at 364 ppm that correlated to a proton resonating at 8.45 ppm which was assigned to the vinyl proton in the CH=N–OH moiety (Figure S13). To reduce the oxime moiety to the aminomethyl group, we investigated various reducing reagents, however, the best results were obtained when 8 equiv of ammonium formate and 6 equiv of zinc dust were used in boiling methanol, which gave *rac*-2-aminomethyl-1-ferrocenecarboxylic acid **13**. The obtained acid was directly transformed into Fmoc-protected **14** in a reaction with an excess of Fmoc-Cl and sodium carbonate as a base in a mixture of water–acetonitrile. Precursor **14** was isolated in 13% overall yield after chromatography on silica. The formation of the desired compound was confirmed by ^1^H and ^13^C{^1^H} NMR spectroscopy.

**Scheme 2:**
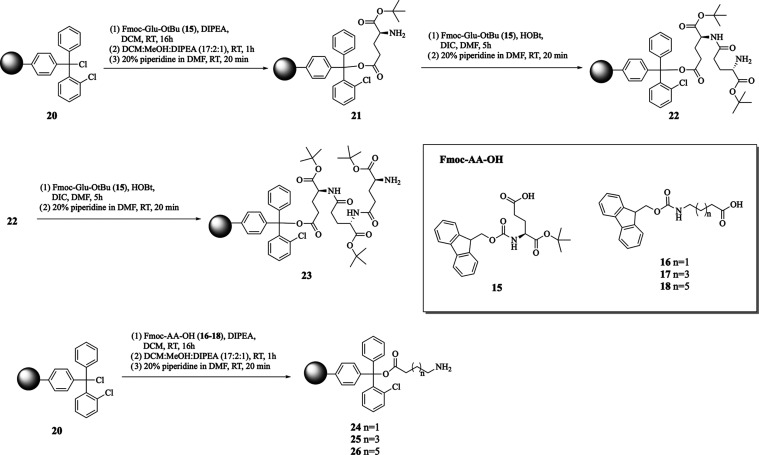
Synthesis of Functionalized Resins **21**–**26**

**Scheme 3:**
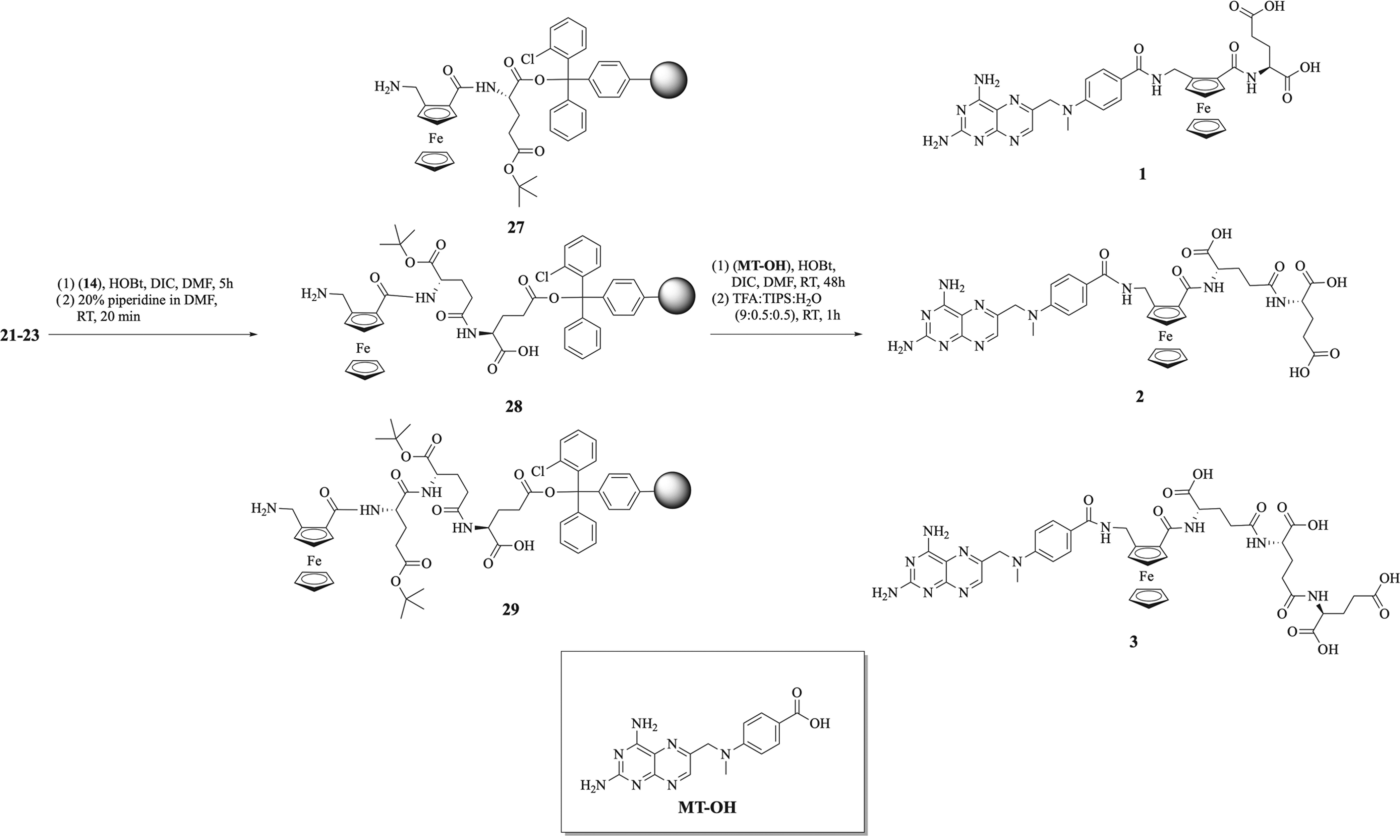
Synthesis of Functionalized Resins **27**–**29** and the Ferrocenyl Conjugates **1**–**3**

**Scheme 4:**
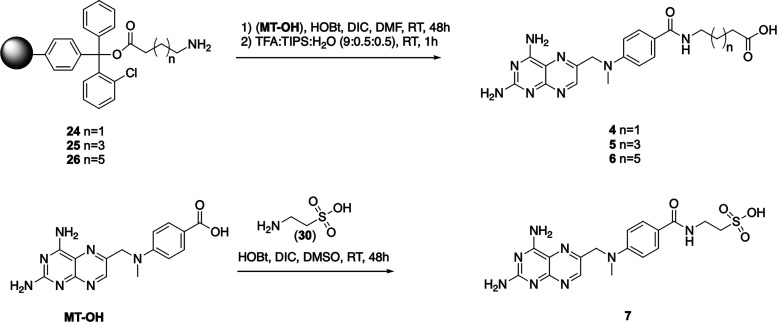
Synthesis of the Conjugates **4**–**7**

The target compounds **1**–**7** were synthesized from di- and tripeptides bound to the resin **24**–**29** or amino acid **30** (Schemes 2 and 3). The required resins were prepared starting from 2-chlorotrityl resin **20** using the same procedure (Scheme 2) that involved first the reaction of 2-chlorotrityl resin with Fmoc-protected amino acids **15**–**18** and diisopropylethylamine (DIPEA) in dichloromethane for 16 h. Next, free positions on 2-chlorotrityl resin were blocked with a capping solution for 1 h, and the Fmoc group was removed in reaction with 20% piperidine in DMF for 20 min, producing monosubstituted resins **21** and **24**–**26**. Further reaction of resin **21** with Fmoc-Glu(O^*t*^Bu)–OH **15** and HOBt and DIC as a coupling agent at RT for 5 h, followed by deprotection with 20% piperidine in DMF, resulted in the formation of resin-dipeptide **22**. Repeating the latter steps of conjugation of **22** with **15** allowed obtaining tripeptide-functionalized resin **23**. The conjugation of resins **21**–**23** with **14** in the presence of HOBt and DIC at RT for 5 h, followed by deprotection with 20% piperidine in DMF, resulted in the formation of the ferrocenyl peptides **27**–**29** bound to resin. The target compounds **1**–**6** were synthesized by conjugation of acid **MT–OH** with resins **24**–**29** and HOBt and DIC at RT for 5 h. Products **1**–**6** were cleaved from the resin with trifluoroacetic acid:triisopropylsilane and water (9:0.5:0.5) for 1 h (Scheme 3). Compound **7** was synthesized directly according to a modified procedure^25^ in the reaction of acid **MT–OH** with taurine **30** and HOBt and DIC as coupling agents (Scheme 4). The obtained products **1**–**7** were isolated by preparative high-performance liquid chromatography (HPLC). The structures of the methotrexate conjugates were confirmed by NMR and HPLC-mass spectrometry (HPLC-MS) analysis. The LC–MS data collected for the ferrocenyl conjugates showed singly and doubly charged ions. For compound **1** these ions were detected at *m*/*z* 696.2 and 348.1 and assigned to [M + H]^+^ and [M + H]^2+^, respectively (Figure S1). Similar results were obtained for **2**, with ions detected at *m*/*z* 825.2 and 412.7 (Figure S2), and for **3**, with ions detected at *m*/*z* 954.5 and 477.3 (Figure S3), assigned to [M + H]^+^ and [M + H]^2+^, respectively. The organic methotrexate conjugates **4**–**6** gave only singly charged ions attributed to [M + H]^+^ (Figures S4–S6). HPLC-MS analysis also confirmed a purity higher than 95% for all target compounds. The formation of amide bonds with the amino acids was confirmed by ^1^H–^15^N HSQC NMR spectroscopy. In all cases, we observed the ^15^N signals at ca. 112–121 ppm indicative of amide bond formation (for the ^1^H–^15^N HSQC spectra see Supporting Information (SI)).

### Dihydrofolate Reductase Inhibitory Activity

**Figure 2:**
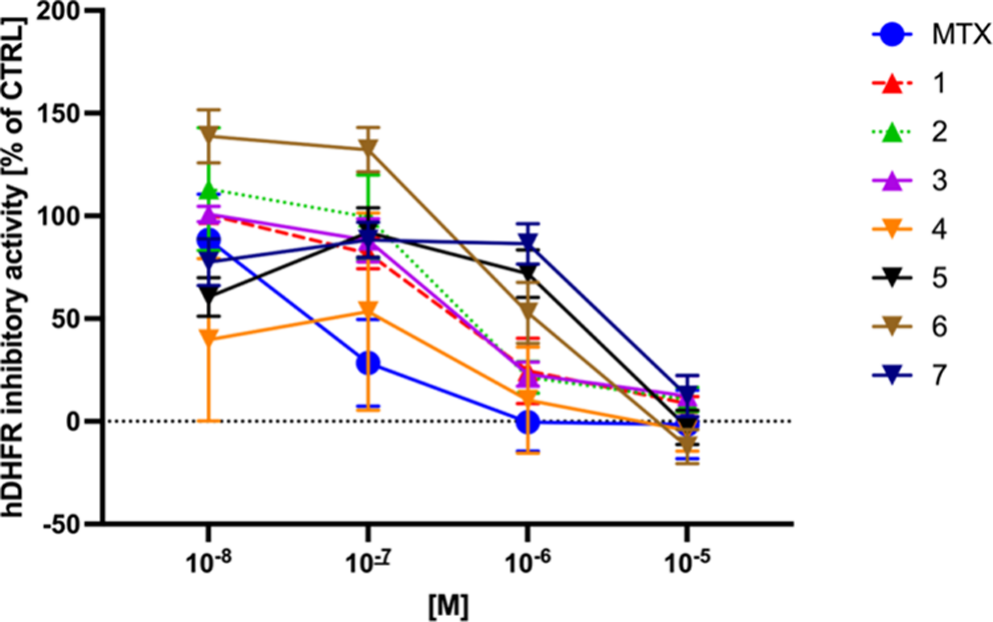
Inhibitory activity of the investigated methotrexate derivatives **1**–**7** toward recombinant hDHFR in comparison to MTX. No modulator was present in the control samples. Data are presented as mean ± SD, *n* = 9 in the case of MTX, 6 for **1**–**4**, and 3 in the case of the other compounds.

We started the evaluation of the biological properties of the investigated MTX derivatives by the assessment of their inhibitory activity toward dihydrofolate reductase. All compounds were applied at concentration of 0.01, 0.1, 1, and 10 μM and methotrexate was used as a reference (Figure 2 and Table 1). Although the enzyme is quite abundant in human cancer cell lines (Puścion, Master of Science thesis), we decided to use the recombinant human DHFR to avoid any concurrent enzymatic activities (the assay is based on β-NADPH oxidation in the presence of dihydrofolic acid and β-NADPH is a cofactor of numerous oxidoreductases).

**Table 1:** DHFR Inhibitory Potential of Methotrexate and its Derivatives^a^

	IC_50_ [μM]
MTX	0.05
**1**	0.38
**2**	0.59
**3**	0.43
**4**	0.01
**5**	1.21
**6**	1.01
**7**	3.03

a IC_50_ values (expressed in μM) were determined based on data presented in Figure 2.

The ferrocenyl conjugates of methotrexate were significantly less active (approximately 1 order of magnitude) than the parent compound and even at the maximum concentration, the DHFR inhibition was incomplete (the residual activity was between 9 and 12%). The length of the poly(glutamate) chain did not correlate with the inhibitory potential of the ferrocenyl MTX derivatives. These results are contrary to observations that the number of glutamate residues enhances the inhibitory potential of MTX, as it was previously demonstrated for various animal liver DHFRs.^28^

To investigate the underlying reason for the reduced activity, the methotrexate derivatives **1**–**7** were docked into the methotrexate binding site of human dihydrofolate reductase (PDB ID: 1U72) in comparison to methotrexate (Tables S1 and S2). The binding site comprises an inner hydrophobic binding site and a peripheral hydrophilic binding pocket with the substituted pteroyl moiety of methotrexate buried deep in the pocket. The docking experiments for methotrexate demonstrated that ChemPLP and Goldscore gave good overlaps with the cocrystallized molecule (Table S1).

**Figure 3:**
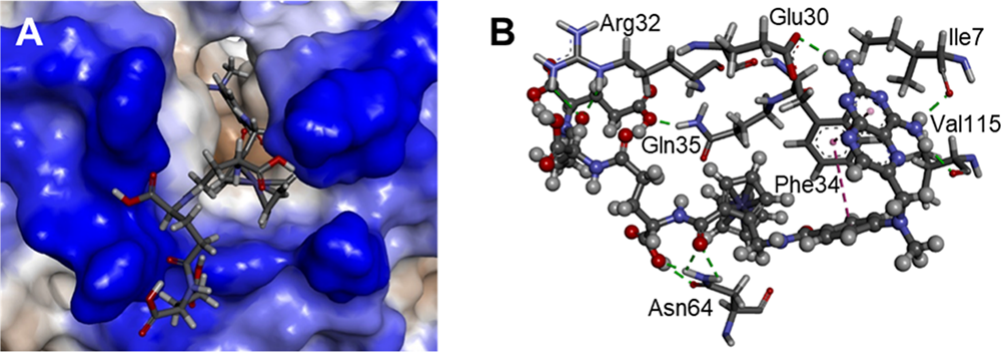
Docked configuration of **3** in the methotrexate binding site of human dihydrofolate reductase as predicted by ChemPLP (A), with **3** occupying the pocket. The protein surface is rendered. Blue depicts a hydrophilic region on the surface, a brown hydrophobic region, and gray shows neutral areas. (B) Hydrogen bonds are shown as green lines between **3** and the amino acids Arg32, Glu30, Gln35, Phe34, Ile7, Asn64, and Val115.

Compounds **1**–**3** can exist as two diastereomers (**1**-P_*R*_, **1**-P_*S*_; **2**-P_*R*_, **2**-P_*S*_; and **3**-P_*R*_, **3**-P_*S*_) due to planar chirality about the substituted ferrocenyl Cp ring, while the chiral centers at the substituents are locked. Modeling showed that a variety of binding modes are possible for the compounds. The two diastereomers of **1**, i.e., **1**-P_*R*_ and **1**-P_*S*_ featured in poses where the ferrocenyl moiety was buried deep in the binding pocket and largely overlapped. All docking scores showed a higher affinity for the former, as was found for the diastereomers of **2**. However, their binding was significantly different with the ferrocene moieties sitting on the protein surface and the pteroyl moieties in the pocket. For compound **3**, isomer **3**-P_*S*_ gave slightly higher docking scores which were however lower than those for the highest-scoring diastereomers of **1** and **2**. The pteroyl substituent of the **3**-P_*R*_ diastereomer showed overlap with that of methotrexate, while that of **3**-P_*S*_ was found on the protein surface. The ferrocenyl groups of both were largely in the same position with the interactions between the compounds and the protein established through π- and hydrogen bonding networks (Figure 3). For example, the **3**-P_*R*_ diastereomer showed π-interactions between the Phe34 phenyl substituent and the aromatic groups of the pteroyl and benzamide moieties of the compound. Also, the pteroyl amino groups of the compound formed hydrogen bonds with the backbone carbonyl oxygen atoms of Val115 and Ile7 as well as the side chain carboxylic oxygen atom of Glu30. In addition, a carbonyl oxygen atom of **3**-P_*R*_ was involved in hydrogen bonds with the side chain and backbone amino groups of Arg32 and Asn64, respectively. Moreover, the hydroxyl groups were part of hydrogen bonds with the backbone amine of Gln35 and the backbone amine and carbonyl moieties of Asn64. Overall, it can be seen that the introduction of the ferrocenyl moieties had a substantial impact on the interaction with the protein which may explain the effects seen, although the docking scores suggest a more effective interaction.

The inhibition pattern of purely organic MTX derivatives was more complex. The shortest side chain derivative **4** significantly reduced the enzymatic activity of DFHR even at the lowest concentration used. On the other side, compounds **5** and **6** were less active, exhibiting inhibitory properties in the micromolar range (Table 1). Rosowsky et al. demonstrated that the distance between the pteroyl and α-carboxyl group of the glutamyl residue is important for MTX interactions with DHFR as increasing the number of γ-aminobutyryl inserts in the side chain resulted in a gradual decrease of inhibitory potency of such “stretched” methotrexate derivatives.^24^ This effect was explained by the reduced availability of the carboxyl group for the invariant arginine residue in the enzyme molecule. However, the introduction of 1 or 2 GABA units increased the DHFR IC_50_ value two- to 3-fold while the effects observed here were at least 2 orders of magnitude more pronounced for much shorter spacers. It can be, however, hypothesized that the astonishing activity of **4** results from the beneficial geometry of this molecule and stronger inhibitor-enzyme interaction. Docking studies showed that the organic molecules **4**–**7** gave similar or lower docking scores compared to methotrexate and they were particularly outperformed by the ferrocenyl derivatives in CHEMPLP and GS. This is surprising given the prominent activity of **4** over the other analogs and crystallization studies may be needed to eventually elucidate this phenomenon. Additionally, poor activity of taurine conjugate **7** indicates the importance of side chain negative charge density and distribution.

### Antiproliferative Activity

To explore the antiproliferative potential of the synthesized compounds we used a set of four cell lines previously employed to study the patterns of multidrug resistance development in the presence of selected chemotherapeutics,^29^ i.e., the colon cancer cell lines CT26.WT (murine) and SW620 (human) and the skin cancer cell lines B16–F10 (murine) and A-431 (human). Along with parental, drug-sensitive cells, we also employed their MTX-resistant variants (denoted with M). To avoid competition of folic acid with the investigated compounds, we decided to use a folate-free medium. However, our attempts to precondition the cells by culturing them in such medium for 2–3 passages before the actual experiment failed as such conditions turned out to be harmful, especially for B16–F10 cells. Therefore, we limited the preconditioning to washing off the normal culture medium and cultivating the cells in folate-free medium for the duration of the experiment (72 h). The results are presented in Table 2.

**Table 2:** Antiproliferative Potential of Methotrexate and its Derivatives towards a Set of MTX-Sensitive and Resistant Cell Lines, as Determined by the Neutral Red Uptake Assay^a^

	A-431	A-431M	B16–F10	B16–F10M	CT26.WT	CT26.WTM	SW620	SW620M
MTX	11.1	22.1	2.0	5.3	0.2	0.7	8.0	32.6
	8.7–14.1	19.6–24.9	1.6–2.5	3.8–7.5	0.1–0.3	0.5–1.0	5.8–11.0	25.3–43.2
		1.99		2.65		3.50		4.08
**1**	62.2	∼79.7	69.0	68.9	20.9	14.7	83.6	≫100
	57.3–68.2		66.0–72.9	65.6–72.7	14.7–30.4	9.6–23.5	78.3–91.2	N/A
		1.28		**1.00**		**0.70**		N/A
**2**	∼85.0	∼93.8	∼98.1	95.2	19.5	16.5	≫100	≫100
	—	—	—	90.6–99.1	15.1–24.1	13.4–20.5	N/A	N/A
		1.10		**0.97**		**0.85**		N/A
**3**	≫100	≫100	≫100	≫100	61.6	47.2	≫100	≫100
	N/A	N/A	N/A	N/A	51.7–75.0	38.3–59.6	N/A	N/A
		N/A		N/A		**0.77**		N/A
**4**	≫100	≫100	≫100	≫100	≫100	≫100	≫100	≫100
	N/A	N/A	N/A	N/A	N/A	N/A	N/A	N/A
		N/A		N/A		N/A		N/A
**5**	≫100	≫100	≫100	≫100	≫100	≫100	≫100	≫100
	N/A	N/A	N/A	N/A	N/A	N/A	N/A	N/A
		N/A		N/A		N/A		N/A
**6**	∼98.6	≫100	≫100	≫100	80	67.5	≫100	≫100
	—	N/A	N/A	N/A	77.5–82.6	63.5–71.9	N/A	N/A
		N/A		N/A		**0.84**		N/A
**7**	∼85.8	∼84.2	∼86.7	∼75.9	∼86.9	65.6	∼83.0	∼96.9
	—	—	—	—	—	60.7–71.0	—	—
		**0.98**		**0.87**		**0.75**		1.17

a IC_50_ values (expressed in μM) were determined based on three independent experiments. 95%-confidence intervals are given in middle rows (please note that due to the log-transformation of the data required to perform IC_50_ calculations, these are asymmetrical) and resistance factors (resistance factor is a ratio of IC_50_ for a given compound for resistant and sensitive line) for respective pairs are reported in lower rows. ≫100 denotes a situation in which IC_50_ values and corresponding confidence intervals could not be determined (<50% viability was not achieved in the concentration range used). Tilde is used to indicate situations when the best-fit value could be found using the software but the confidence intervals could not be precisely calculated (which is indicated by “—” sign).

The investigated compounds exerted at best moderate antiproliferative effects. Of the ferrocenyl MTX derivatives, only **1** was potent enough to significantly influence the proliferation of all cell lines studied although it was 5- to 10-fold less active than the parent compound. Effects of its diglutamyl derivative **2** were even less pronounced while triglutamyl **3** was effective toward the most MTX-sensitive CT26.WT and CT26.WTM cells only. These effects are contrary to those obtained in the enzymatic assay, suggesting that the bulkiness of the compound may limit its entry into the cell. This is not unexpected as it is known that poly(glutamylated) folates have to be converted to the respective monoglutamyl forms before being absorbed by the cell.^30^ Surprisingly, the most powerful DHFR inhibitor **4** completely lacked antiproliferative potential in the cell-based assay, and of the purely organic series only **6** and **7** had some mild effects on cell viability. However, it must be emphasized, that despite the relatively low activity, the observed antiproliferative effects were virtually independent of the MTX-resistance status of the cells. The differences in IC_50_ values for active compounds and sensitive and resistant cells were not statistically significant as corresponding confidence intervals overlapped or the direction of effect was reverted (as in the case of CT26.WT and CT26.WTM cells and compounds **6** and **7**) which was also accentuated by resistance factor values close to 1.

The cell lines differed significantly in terms of response to the investigated compounds with CT26.WT is the most susceptible and SW620 is the least sensitive. Therefore, we focused on CT26.WT and its MTX-resistant counterpart as research models in further studies.

### Cell Cycle Modulatory Effects

**Figure 4:**
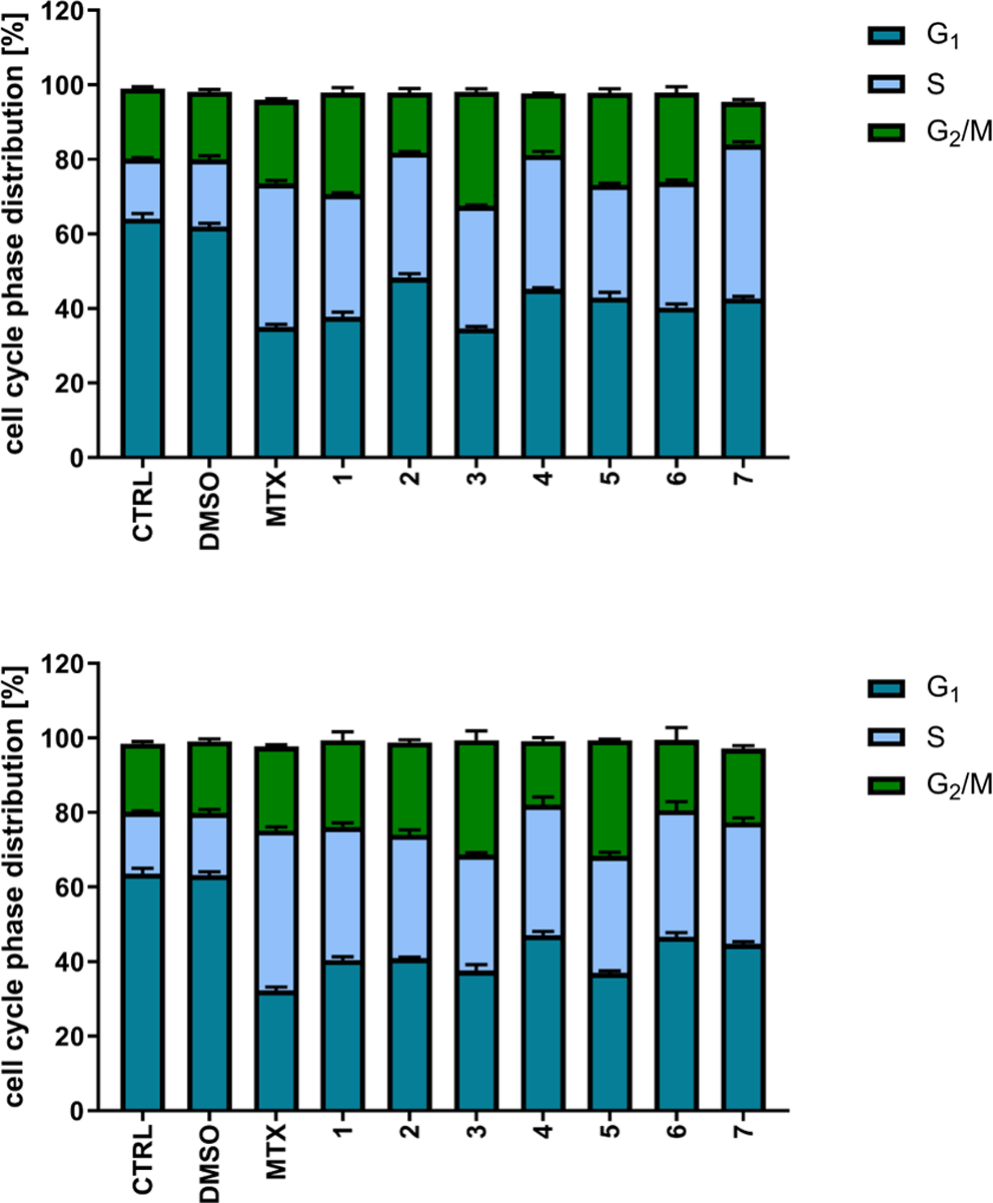
Effects of the investigated ferrocenyl methotrexate derivatives **1**–**3** on cell cycle phase distribution in CT26.WT cells. Upper panel −24 h exposure, lower panel −72 h exposure. Data are presented as mean ± SD, *n* = 3.

To confirm the mode of action of the most active ferrocenyl MTX derivatives, we incubated CT26.WT cells for 24 and 72 h with the investigated compounds at the low concentration (equal to IC_75_ for MTX) and determined cell cycle phase distribution (Figure 4). In control samples, over 64% of cells remained in the G_1_ phase, about 16% of the cells were found in the S phase and 18–19% were dividing (G_2_/M). The effect of solvent (DMSO) was negligible. MTX exposure resulted in the reduction of the G_1_ cell fraction to 35% after 24 h and 32% after 72 h, and an increase in the S phase fraction to, respectively, 38 and 43%, with approximately 22–23% of mitotic cells. A similar situation was observed for investigated MTX derivatives, with approximately 40–49% of G_1_ cells, 30–40% of S cells, and 11–24% of G_2_/M cells, irrespective of the compound. These results demonstrate that the antifolate mode of action manifesting as stopping the cell division in the S phase due to lack of DNA building blocks is preserved in MTX analogues.

### Direct Effects on Abcc1 Activity

**Figure 5:**
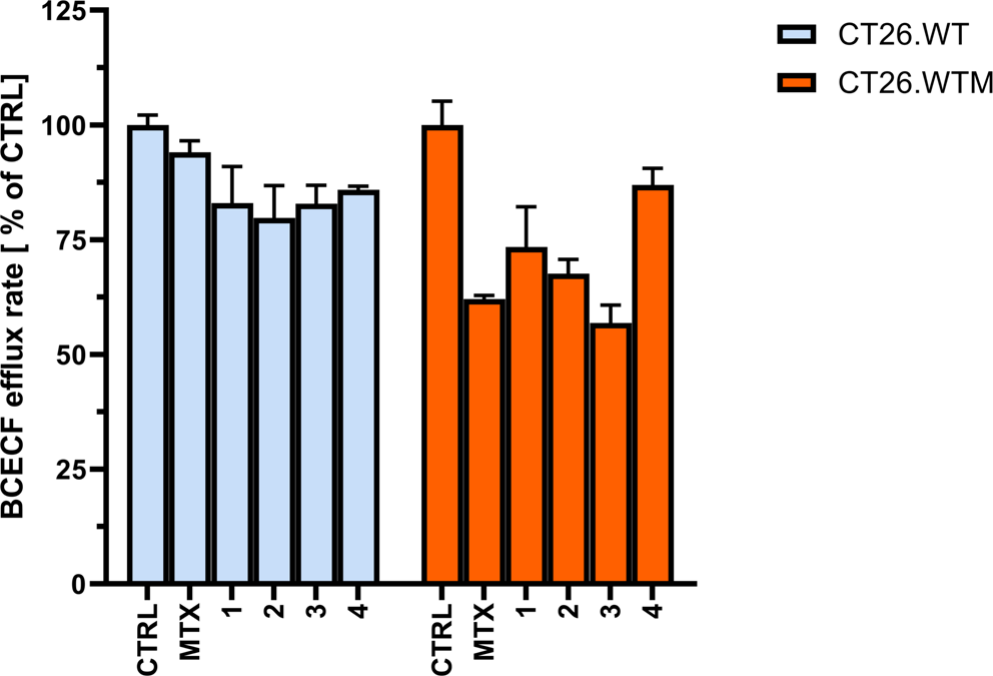
Effects of the investigated methotrexate derivatives **1**–**4** (100 μM) on Abcc1 activity measured as the BCECF efflux. Data of a representative experiment. The initial efflux rate is presented along with standard error.

ABCC1/Abcc1 mediates drug resistance by eliminating drugs or their metabolites from the cytoplasm, thus diminishing their effective concentration. To inspect whether the effects of the investigated compounds observed in the proliferation assay are indeed transporter-dependent, we performed a real-time efflux assay (Figure 5). The cells were loaded with a nonfluorescent acetoxymethyl ester of 2′,7′-bis(2-carboxyethyl)-5-(and-6)-carboxyfluorescein (BCECF-AM) which is intracellularly hydrolyzed to brightly fluorescent BCECF. The latter, as a large organic anion, is unable to cross the plasma membrane by diffusion and is retained in the cytoplasm. If ABCC1/Abcc1 is active in the cell, the dye is actively exported to the external milieu and the cell fluorescence decreases with time.^31^ If ABCC1/Abcc1 activity is impeded by an inhibitor (or a competing substrate), the rate of intracellular fluorescence reduction is much lower. Thus, this assay allows for the detection of direct interactions between a potential modulator and the transporter molecule.

To ensure the intracellular concentration of MTX derivatives, the cells were preincubated with 100 μM of the investigated compounds for 1 h before the actual experiment. The compounds were also present during the measurement. Despite the high concentration used, the exposure time was too short for the compounds to exert any detrimental effects on the cells taking into account their mode of action. Ferrocenyl MTX derivatives inhibited Abcc1 activity to an extent comparable with MTX (approximately 55–73 vs 62% of control, respectively). The GABA conjugate **4** was almost inactive in terms of modulatory effects on the protein. Considering the combined results of the proliferation and transport assays, it may be inferred that the direct interaction of the investigated compounds with the multidrug transporter may contribute to overcoming the MTX-resistance phenomenon.

## Conclusions

We report two series of methotrexate derivatives differing in their side chain composition. Of the ferrocenyl conjugates, the number of glutamyl moieties had no evident effect on DHFR inhibitory potency, while was inversely proportional to their antiproliferative effects. Of the amino acid conjugates, the GABA-decorated pteroyl conjugate was an even more effective DHFR inhibitor than MTX, while it had no effects on intact cells. The ferrocenyl derivatives of methotrexate were able to cross the drug resistance barrier formed by the ABCC1/Abcc1 protein. They were also able to directly interact with the transporter molecule. The combined results emphasize the importance of cellular folate uptake/export systems for the biological activity of antifolates.

## Materials and Methods

### General

All reactions were performed under an argon atmosphere using the standard Schlenk technique. Solvents were dried before use by distillation from sodium-benzophenone (tetrahydrofuran) or calcium hydride (dichloromethane). Reagents (Sigma Aldrich or Fluorochem) were used as received. One-dimensional (1D) and two-dimensional (2D) NMR spectra were recorded on a Bruker Avance III 600 MHz spectrometer at 294 K with spectrometer frequencies of 600.26 MHz for ^1^H and 150.94 MHz for ^13^C or Bruker Avance Neo 600 MHz spectrometer equipped with N2-cryoprobe with spectrometer frequencies of 600.1 MHz for ^1^H and 150.0 MHz for ^13^C. Chemical shifts were referenced to residual peaks in DMSO-*d*_6_ δ = 2.51 ppm for ^1^H and δ = 39.5 ppm for ^13^C. Chemical shifts are given in ppm and coupling constants in Hertz (Hz).

The target compounds **1**–**7** were isolated using a Shimadzu HPLC system on a semiprep column (Phenomenex PFP 150 × 21.6 mm, 5 μm, flow rate 25 mL·min^–1^) with a diode array detector (SPD-M20A) and a gradient of A:B starting from 0 to 100% of B within 30 min, where A is 0.1% TFA in H_2_O and B is 0.1% TFA in MeCN. The purity of most of the compounds was ≥95% as measured by HPLC-MS analysis on an analytical Phenomenex XB-C18 column (50 × 4.6 mm, 1.7 μm) with a mobile phase flow of 0.4 mL·min^–1^ using a Shimadzu Nexera XR system equipped with SPD-M40 and LC–MS-2020 detectors using a gradient of phase A (water + 0.01% of HCOOH) and phase B (methanol + 0.01% of HCOOH) starting from 5% of B (*t* = 0 min), then 90% of B (*t* = 3 min), hold time (*t* = 4 min), 5% of B (*t* = 4.5 min), stop analysis time at *t* = 7 min. **MT–OH** was synthesized according to a reported procedure.^32^ Compound **7** was synthesized according to a modified procedure.^25^

### Synthesis of *rac*-2-Formyl-1-ferrocenecarboxylic Acid **11**

This compound was synthesized according to a modified known procedure.^33^ 17.8 mL (24.9 mmol) of *sec*-BuLi (*c* = 1.4 M in cyclohexane) was added at −78°C within 10 min to a solution of 6.15 g (22.6 mmol) of **9** in 90 mL of anhydrous THF. The reaction mixture was warmed up to −10°C and stirred for 1 h. The resulting suspension was cooled down to −78°C and dry CO_2_ was bubbled through the reaction mixture for 1.5 h. During the bubbling of CO_2_, the reaction mixture was allowed to warm up to room temperature. After finishing bubbling, a few pieces of dry ice (ca. 50 g) were added to the reaction mixture very carefully to complete the reaction. The resulting slurry was dissolved in 50 mL of THF and 100 mL of 1 M solution of hydrochloric acid was carefully added and stirred for 30 min. The reaction mixture was basified using sodium carbonate until pH = 10 and diluted with dichloromethane. The organic phase was washed twice with dichloromethane (2 × 100 mL) and the combined organic layers were discarded. An aqueous layer was acidified to pH = 1 using concentrated hydrochloric acid and the product was extracted with dichloromethane (3 × 100 mL). The combined organic layers were washed with brine, dried over anhydrous sodium sulfate, filtered, and evaporated to dryness, giving **11** as a dark red solid in 83% yield (4.85 g). ^1^H NMR (CDCl_3_) δ = 12.98 (br. s, 1H, COOH), 9.95 (s, 1H, CHO), 5.63 (s, 1H, Cp(H-3)), 4.98–4.96 (m, 2H, Cp(H4,5)), 4.44 (s, 5H, Cp′). ^13^C{^1^H} NMR (CDCl_3_) δ = 197.2 (CHO), 169.5 (COOH), 80.5 (C3), 77.9 (C4 or 5), 76.4 (C4 or 5), 75.4 (C1), 73.3 (C2), 72.4 (Cp′). The NMR spectra were identical to those reported in the literature.^27^

### Synthesis of 2-(Hydroxyiminomethyl)-1-ferrocenecarboxylic Acid **12**

6.40 g (92.1 mmol) of hydroxylamine hydrochloride followed by 17.1 mL (101 mmol) of triethylamine were added to a solution of 7.93 g (30.7 mmol) of **11** in 154 mL of absolute ethanol at room temperature under argon atmosphere and the resulting mixture was stirred at RT for 16 h. Then, the reaction mixture was quenched by adding 100 mL of 1 M hydrochloric acid and additionally acidified using concentrated hydrochloric acid to pH = 1. The crude product was extracted with dichloromethane (3 × 50 mL) and combined organic solutions were washed with brine, dried over anhydrous sodium sulfate, filtered, and evaporated to dryness. The crude product was purified by flash chromatography on silica gel using a gradient of methanol in dichloromethane (starting from 2 to 10% methanol) and afforded the desired **12** as a dark red solid in 92% yield (7.65 g) which was used in the next step. An analytically pure sample of **12** was obtained by chromatography on silica using a gradient of methanol in dichloromethane starting from 1 to 3% of methanol. ESI-MS calculated for C_12_H_12_FeNO_3_*m*/*z* = 274.0 [M + H]^+^ found *m*/*z* = 274.1 [M + H]^+^. Elemental analysis calculated for C_12_H_11_FeNO_3_ C 52.8, H 4.1, N 5.1%, found C 52.9; H 4.3, N 5.2%. ^1^H NMR (DMSO-*d*_6_) δ = 12.52 (br. s, 1H, COOH), 10.82 (s, 1H, CH=NOH), 8.45 (s, 1H, CH=NOH), 4.862 (s, 1H, Cp), 4.858 (s, 1H, Cp), 4.59 (t, 1H, *J* = 2.4 Hz, Cp), 4.23 (s, 5H, Cp′). ^13^C{^1^H} NMR (DMSO-*d*_6_) δ = 172.1 (COOH), 146.8 (CH=NOH), 79.1 (s, Cp_ipso_), 72.1 (Cp), 71.2 (Cp), 70.6 (Cp′), 70.4 (Cp_ipso_), 68.8 (Cp); ^15^N (DMSO-*d*_6_) δ = 364 (based on ^1^H–^15^N HMBC).

### Synthesis of *rac*-2-(((((9*H*-Fluoren-9-yl)methoxy)carbonyl)amino)methyl)-1-ferrocenecarboxylic Acid 14

To a solution of 2.05 g (7.51 mmol) of oxime **12** in 15 mL of anhydrous methanol at RT under argon atmosphere 2.95 g (45.1 mmol) of powdered zinc followed by 3.79 g (60.1 mmol) of ammonium formate were added and the resulting mixture was refluxed for 16 h. Next, the reaction mixture was filtered through Celite and evaporated to dryness giving crude **13**. Next, 2.33 g (9.01 mmol) of Fmoc chloride followed by 4.0 g (37.5 mmol) of sodium carbonate was added to the solution of crude **13** in 50 mL of water and 50 mL of acetonitrile and the resulting solution was stirred at room temperature for 20 h. Then, 1.0 mL of DIPEA and the second portion of 1.0 g (3.86 mmol) of Fmoc chloride were added and the resulting mixture was stirred for an additional 20 h. The reaction was quenched with 50 mL of 1 M hydrochloric acid solution and the crude product was extracted with dichloromethane (3 × 50 mL). Chromatography on silica gel using the gradient of methanol in dichloromethane (starting from 2% up to 10% methanol) afforded **9** in 13% yield (0.485 g) as a yellow solid. ESI-MS calculated for C_27_H_24_FeNO_4_*m*/*z* = 482.1 [M + H]^+^ found *m*/*z* = 482.3 [M + H]^+^. ^1^H NMR (DMSO-*d*_6_) δ = 12.24 (br. s, 1H, COOH), 7.89 (d, *J* = 7.5 Hz, 2H, CH_Ar_), 7.73 (t, *J* = 6.7 Hz, 2H, CH_Ar_), 7.54 (t, *J* = 5.9 Hz, 1H, NH), 7.42 (t, *J* = 7.4 Hz, 2H, CH_Ar_), 7.35–7.32 (m, 2H, CH_Ar_), 4.64 (s, 1H, Cp), 4.42–4.32 (m, 5H, CH_2_NHCOOCH_2_ (3H) and Cp (2H)), 4.24 (t, *J* = 6.8 Hz, 1H, CH_2_CH_Fmoc_), 4.18–4.13 (m, 1H, CH_2_NH) overlapped with 4.16 (s, 5H, Cp′); ^13^C{^1^H} NMR (DMSO-*d*_6_) δ = 172.8 (COOH), 156.2 (NHCO), 143.9 (2 × C_Ar_), 140.7 (C_Ar_), 127.6 (CH_Ar_), 127.0 (2 × CH_Ar_), 125.2 (2 × CH_Ar_), 120.1 (CH_Ar_), 88.8 (Cp_ipso_), 72.1, 71.4 (Cp), 70.5 (Cp), 70.1 (Cp′), 70.0, 69.2 (Cp_ipso_), 68.9 (Cp), 65.3 (CH_2_NH), 46.8 (CH), 38.6 (OCOCH_2_).

### General Procedure A—Conjugation of the Resin **20** with Fmoc-Protected Amino Acids **15**–**18**—Synthesis of Resins **21**, **24**–**26**

2.5 equiv of DIPEA was added to a slurry of the amino acid (2.5 equiv) in DCM and the obtained mixture was stirred until the amino acid was completely dissolved. The resulting solution was immediately added to the 2-chlorotrityl resin, pretreated with DCM for 30 min, and the mixture was reacted for 16 h. After removal of the reagents by suction, the obtained resin was washed with DCM and treated with the capping mixture (DCM-MeOH-DIPEA, 17:2:1) for 1 h. Then the solution was removed, the resin was washed with DCM and DMF and the Fmoc group was removed by treating the resin with 20% piperidine in DMF (twice, each cycle 20 min). The obtained resin was washed 3 times with DMF, DCM, and methanol and dried under vacuum for 1 h.

**Resin 21** was synthesized according to **General Procedure A** starting from 1.0 g (1.6 mmol) of 2-chlorotrityl resin **20**, 1.7 g (4.0 mmol) of **15**, 517 mg (700 μL, 4.0 mmol) of DIPEA, and 15 mL of DCM.

**Resin 24** was synthesized according to **General Procedure A** starting from 0.25 g (0.4 mmol) of 2-chlorotrityl resin **20**, 0.325 g (1.0 mmol) of **16**, 129 mg (175 μL, 1.0 mmol) of DIPEA, and 5 mL of DCM.

**Resin 25** was synthesized according to **General Procedure A** starting from 0.50 g (0.8 mmol) of 2-chlorotrityl resin **20**, 0.706 g (2.0 mmol) of **17**, 258 mg (350 μL, 2.0 mmol) of DIPEA, and 10 mL of DCM.

**Resin 26** was synthesized according to **General Procedure A** starting from 0.25 g (0.4 mmol) of 2-chlorotrityl resin **20**, 0.381 g (1.0 mmol) of **18**, 129 mg (175 μL, 1.0 mmol) of DIPEA, and 5 mL of DCM.

### General procedure B—Synthesis of Peptides: Conjugation of Resin **21** with Amino Acids—Preparation of Resins **22**–**23** and **27**–**29**

DMF solutions containing 2.5 equiv of conjugated amino acids, HOBt and DIC were added to resin **21** (pretreated with DCM for 30 min) and the obtained mixture was reacted at RT for 4 h. Then, the excess reagents were removed by suction, the resin was washed 3 times with DMF and the Fmoc group was removed by treating the resin with 20% piperidine in DMF (twice, each cycle 20 min). The obtained resin was washed 3 times with DMF, DCM, and methanol and dried under vacuum for 1 h.

**Resin 22** was synthesized according to **General Procedure B** starting from 3.18 g of resin **21**, 5.11 g (12.0 mmol) of **15**, 1.62 g (12.0 mmol) of HOBt, and 1.52 g (1.88 mL, 12.0 mmol) of DIC in 10 mL of DMF.

**Resin 23** was synthesized according to **General Procedure B** starting from 1.62 g of resin **22**, 2.72 g (6.4 mmol) of **15**, 0.865 g (6.4 mmol) of HOBt, and 0.808 g (1.0 mL, 6.4 mmol) of DIC in 10 mL of DMF.

**Resin 27** was synthesized according to **General Procedure B** starting from 0.22 g of resin **21**, 0.385 g (0.80 mmol) of **14**, 0.108 g (0.80 mmol) of HOBt, and 101 mg (125 μL, 0.80 mmol) of DIC in 1.0 mL of DMF.

**Resin 28** was synthesized according to **General Procedure B** starting from 0.22 g of resin **22**, 0.385 g (0.80 mmol) of **14**, 0.108 g (0.80 mmol) of HOBt, and 101 mg (125 μL, 0.80 mmol) of DIC in 1.0 mL of DMF.

**Resin 29** was synthesized according to **General Procedure B** starting from 0.22 g of resin **23**, 0.385 g (0.80 mmol) of **14**, 0.108 g (0.80 mmol) of HOBt, and 101 mg (125 μL, 0.80 mmol) of DIC in 1.0 mL of DMF.

### General Procedure C—Conjugation of Resins **24**–**29** with MT–OH—Preparation of Methotrexate Derivatives **1**–**6**

The respective resin was pretreated with DCM for 30 min, the solvent was removed by suction and the resin was washed with DMF and DMSO. Then, a solution of 2.5 equiv of **MT–OH** and 2.5 equiv of HOBt in DMF followed by 2.5 equiv of DIC were added to the obtained resin. The obtained mixture was reacted at RT for 48 h and the excess of the reagents was removed by suction. The obtained resin was washed with DMF, DCM, and methanol and dried under vacuum for 1 h. Next, the products were cleaved off the resin by treatment with a mixture of TFA-TIPS-H_2_O (9:0.5:0.5) for 1 h. Next, the solution was evaporated, the residue dissolved in DMSO and the compounds were purified by HPLC. Fractions containing the product were combined and freeze-dried.

#### Synthesis of **1**

Compound **1** (31 mg) was synthesized according to General Procedure B starting from **resin 27** and 286 mg (0.88 mmol) of **MT–OH**, 119 mg (0.88 mmol) of HOBt, and 111 mg (137 μL, 0.880 mmol) of DIC in 2.00 mL DMF. *R*_f_ (HPLC) τ = 4.62 min, purity 98.4%; ESI-MS (*m*/*z*) calculated for C_32_H_34_FeN_9_O_6_*m*/*z* 696.2 [M + H]^+^, found *m*/*z* 696.2 [M + H]^+^ and 348.1 [M + H]^2+^. ^1^H NMR (600 MHz, DMSO-*d*_6_) δ = 13.02–12.18 (br m, COOH), 9.20 (br s, 1H, NH), 9.0 (br s, 1H, NH), 8.72 (s, 1H, CH_Ar_), 8.56 (t, *J* = 5.9 Hz, 1H, NH), 8.47 (t, *J* = 6.1 Hz, 1H, NH), 8.18 (d, *J* = 7.8 Hz, 0.5H, NH), 8.00 (d, *J* = 7.8 Hz, 0.5H, NH), 7.71 (t, *J* = 8.4 Hz, 2H, 1,4-C_6_H_4_), 7.52 (br. s, 1H, NH), 6.84–6.83 (m, 2H, 1,4-C_6_H_4_), 4.94 (br s, 0.5H, Cp), 4.91 (br s, 0.5H, Cp), 4.86 (s, 2H, CH_2_–N(CH)_3_), 4.53 (dt, *J* = 6.3, 15.1 Hz, 1H, CH_2_), 4.45–4.34 (m, 3H, CH_2_ and CH), 4.27–4.26 (m, 1H, Cp), 4.22 (s, 2.5H, Cp′), 4.10 (s, 2.5H, Cp′), 3.24 (s, 3H, CH_2_–N(CH)_3_), 2.43 (t, *J* = 7.3 Hz, 1H, CH_2_), 2.37–2.34 (m, 1H, CH_2_), 2.17–2.21 (m, 1H, CH_2_), 1.99–1.93 (m, 1H, CH_2_).

#### Synthesis of **2**

Compound **2** (56 mg) was synthesized according to General Procedure B starting from **resin 28** and 286 mg (0.88 mmol) of **MT–OH**, 119 mg (0.88 mmol) of HOBt and 111 mg (137 μL, 0.880 mmol) of DIC in 3.00 mL DMF. *R*_f_ (HPLC) τ = 4.7 min, purity 98.8%; ESI-MS (*m*/*z*) calculated for C_37_H_41_FeN_10_O_9_*m*/*z* 825.2 [M + H]^+^, found *m*/*z* 825.2 [M + H]^+^ and 412.7 [M + H]^2+^. ^1^H NMR (600 MHz, DMSO-*d*_6_) δ = 13.00–12.16 (br m, COOH), 9.23 (br s, 1H, NH), 9.03 (br s, 1H, NH), 8.72 (s, 1H, CH_Ar_), 8.56 (t, *J* = 6.0 Hz, 1H, NH), 8.47 (t, *J* = 5.9 Hz, 1H, NH), 8.28 (d, *J* = 7.5 Hz, 0.5 H, NH), 8.18 (d, *J* = 7.8 Hz, 0.5 H, NH), 8.16 (d, *J* = 7.8 Hz, 0.5 Hz, NH), 8.10 (d, *J* = 7.6 Hz, 0.5 Hz, NH), 7.72 (d, *J* = 8.8 Hz, 1H, 1,4-C_6_H_4_), 7.71 (d, *J* = 8.8 Hz, 1H, 1,4-C_6_H_4_), 7.51–7.45 (m, 1H, 1,4-C_6_H_4_), 6.84 (d, *J* = 9.1 Hz, 1H, 1,4-C_6_H_4_), 6.84 (d, *J* = 9.1 Hz, 1H, 1,4-C_6_H_4_), 4.95–4.94 (m, 0.5 H, Cp), 4.90–4.89 (m, 0.5H, Cp), 4.86 (s, 2H, CH_2_–N(CH)_3_), 4.54–4.49 (m, 1H, CH_2_), 4.41–4.31 (m, 3H, CH_2_ and CH), 4.27–4.22 (m, 5 H, Cp′ and CH), 4.16 (s, 2H, Cp′), 3.24 (s, 3H, CH_2_–N(CH)_3_), 2.38–2.32 (m, 4H, CH_2_), 2.11–2.07 (m, 1 H, CH_2_), 2.00–1.92 (m, 2H, CH_2_), 1.78–1.74 (m, 1H, CH_2_).

#### Synthesis of **3**

Compound **3** (15 mg) was synthesized according to General Procedure B starting from **resin 29** and 286 mg (0.88 mmol) of **MT–OH**, 119 mg (0.88 mmol) of HOBt and 111 mg (137 μL, 0.880 mmol) of DIC in 3.00 mL DMF. *R*_f_ (HPLC) τ = 4.87 min, purity 90.1%; ESI-MS (*m*/*z*) calculated for C_42_H_48_FeN_11_O_12_*m*/*z* 954.3 [M + H]^+^, found *m*/*z* 954.5 [M + H]^+^ and 477.3 [M + H]^2+^. ^1^H NMR (600 MHz, DMSO-*d*_6_) δ 12.52 (br s, COOH), 9.22 (br s, 1H, NH), 9.02 (br s, 1H, NH), 8.72 (s, 1H, CH_Ar_), 8.55 (t, *J* = 6.0 Hz, 0.5H, NH), 8.46 (t, *J* = 5.9 Hz, 0.5H, NH), 8.31 (d, *J* = 7.5 Hz, 0.5H, NH), 8.22–8.14 (m, 1.5 H, NH), 8.10 (d, *J* = 7.6 Hz, 1H, NH), 8.09 (d, *J* = 8.5 Hz, 1H, NH), 7.72 (d, *J* = 8.8 Hz, 1H, 1,4-C_6_H_4_), 7.71 (d, *J* = 8.8 Hz, 1H, 1,4-C_6_H_4_), 7.42 (br s, 1H, NH), 6.85–6.83 (m, 2H, 1,4-C_6_H_4_), 4.94 (br s, 0.5H, Cp), 4.90 (br s, 0.5H, Cp), 4.86 (s, 2H, CH_2_–N(CH)_3_), 4.55–4.50 (m, 1H, CH_2_), 4.41–4.31 (m, 3H, CH_2_ and CH), 4.27–4.26 (m, 2H, Cp), 4.22 (s, 3H, Cp′), 4.21–4.17 (m, 6H, Cp′ and CH), 3.24 (s, 3H, CH_2_–N(CH)_3_), 2.39–2.17 (m, 6H, CH_2_), 2.10–2.07 (m, 1H, CH_2_), 2.00–1.91 (m, 4H, CH_2_), 1.78–1.72 (m, 2H, CH_2_).

#### Synthesis of **4**

Compound **4** (56 mg) was synthesized according to General Procedure B starting from **resin 24** and 324 mg (1.00 mmol) of **MT–OH**, 135 mg (1.00 mmol) of HOBt, and 126 mg (156 μL, 1.00 mmol) of DIC in 10.00 mL DMF. *R*_f_ (HPLC) τ = 3.52 min; purity 95.2%; ESI-MS (*m*/*z*) calculated for C_19_H_23_N_8_O_3_*m*/*z* 411.2 [M + H]^+^, found *m*/*z* 411.1 [M + H]^+^. ^1^H NMR (600 MHz, DMSO-*d*_6_) δ 12.94 (br s, 1H), 12.01 (s, 1H), 9.12 (br s, 1 H, NH), 8.93 (br s, 1H, NH), 8.70 (s, 1H, CH_Ar_), 8.11 (t, *J* = 5.6 Hz, 1H, NHCO), 7.69 (d, *J* = 8.9 Hz, 2H, 1,4-C_6_H_4_), 7.38 (br s, 1H, NH), 6.80 (d, *J* = 9.0 Hz, 2H, 1,4-C_6_H_4_), 4.85 (s, 2H, CH_2_–N(CH)_3_), 2.23 (s, 3H, CH_2_–N(CH)_3_), 3.21 (q, *J* = 6.3 Hz, 2H, CH_2_), 2.23 (t, *J* = 7.4 Hz, 2H, CH_2_), 1.72–1.68 (m, 2H, CH_2_).

#### Synthesis of **5**

Compound **5** (33 mg) was synthesized according to General Procedure B starting from **resin 25** and 648 mg (2.00 mmol) of **MT–OH**, 270 mg (2.00 mmol) of HOBt, and 252 mg (313 μL, 2.00 mmol) of DIC in 7.0 mL DMF. *R*_f_ (HPLC) τ = 3.92 min; purity 98.0%; ESI-MS (*m*/*z*) calculated for C_21_H_27_N_8_O_3_*m*/*z* = 439.2 [M + H]^+^, found *m*/*z* = 439.3 [M + H]^+^. ^1^H NMR (600 MHz, DMSO-*d*_6_) δ = 11.91 (br s, 1H), 8.64 (s, 1H, CH_Ar_), 8.61 (br s, 1H, NH),. 8.40 (br s, 1H, NH), 8.07 (t, *J* = 5.6 Hz, 1H, NHCO), 7.68 (d, *J* = 8.9 Hz, 2H, 1,4-C_6_H_4_), 7.26 (br s, 1H, NH), 6.80 (d, *J* = 9.0 Hz, 2H, 1,4-C_6_H_4_), 4.82 (s, 2H, CH_2_–N(CH)_3_), 3.22 (s, 3H, CH_2_–N(CH)_3_), 3.18 (q, *J* = 6.6 Hz, 2H, CH_2_), 2.19 (t, *J* = 7.38 Hz, 2H, CH_2_), 1.52–1.44 (m, 4H, CH_2_), 1.30–1.24 (m, 2H, CH_2_).

#### Synthesis of **6**

Compound **5** (65 mg) was synthesized according to General Procedure B starting from **resin 26** and 324 mg (1.00 mmol) of **MT–OH**, 135 mg (1.00 mmol) of HOBt, and 126 mg (156 μL, 1.00 mmol) of DIC in 10.00 mL DMF. *R*_f_ (HPLC) τ = 5.10 min; purity 94.7%; ESI-MS (*m*/*z*) calculated for C_23_H_31_N_8_O_3_*m*/*z* = 467.2 [M + H]^+^, found *m*/*z* = 467.4 [M + H]^+^. ^1^H NMR (600 MHz, DMSO-*d*_6_) δ 11.09 (br s, 1H), 9.19 (br s, 1H, NH), 8.99 (br s, 1H, NH), 8.70 (s, 1H, CH_Ar_), 8.49 (br s, 1H, NH), 8.05 (t, *J* = 5.6 Hz, 1H, NHCO), 7.68 (d, *J* = 9.0 Hz, 2H, 1,4-C_6_H_4_), 7.44 (br s, 1H, NH), 6.80 (d, *J* = 9.0 Hz, 2H, 1,4-C_6_H_4_), 4.85 (s, 2H, CH_2_–N(CH)_3_), 3.23 (s, 3H, CH_2_–N(CH)_3_), 3.18 (q, *J* = 6.6 Hz, 2H, CH_2_), 1.50–1.45 (m, 4H, CH_2_), 1.26 (br s, 6H, CH_2_).

### Dihydrofolate Reductase Assay

The recombinant human dihydrofolate reductase (DFHR) was purchased from R&D (Bio-Techne, Minneapolis, Minnesota) and its activity in the presence of the synthesized compounds was assayed as per the manufacturer’s instructions. The assay buffer consisted of 50 mM 2-(*N*-morpholino)ethanesulfonic acid (MES), 25 mM tris(hydroxymethyl)aminomethane (Tris), 100 mM NaCl, 25 mM ethanolamine, 2 mM dithiotreitol, 0.1 mM dihydrofolic acid and 0.125 mM β-NADPH. The final enzyme concentration was 1 μg/mL. The absorbance at 340 nm was monitored over 15 min at 37°C. The initial rate of absorbance decrease over time (up to 5 min) was considered as the measure of DHFR activity as per the manufacturer’s instructions.

### Molecular Modeling

Compounds **1**–**7** were docked into the crystal structure of human dihydrofolate reductase (PDB ID: 1U72, resolution 1.90 Å),^34^ which was obtained from the Protein Data Bank (PDB).^35,36^ For **1**–**3**, both diastereomers with regard to the difference in planar chirality about the substituted ferrocenyl Cp ring were investigated, i.e., **1**-P_*R*_, **1**-P_*S*_, **2**-P_*R*_, **2**-P_*S*_, **3**-P_*R*_, **3**-P_*S*_. Scigress version FJ 2.6^37^ was used to prepare the crystal structure for docking, i.e., hydrogen atoms were added and the cocrystallized methotrexate was removed. The center of the binding site was defined as the carbon atom on the phenyl ring adjacent to the carbonyl carbon of methotrexate (*x* = 32.139, *y* = 18.3040, *z* = 0.049) with a radius of 10 Å. The scoring functions GoldScore (GS)^38^ and ChemScore (CS),^39,40^ ChemPLP^41^ and Astex statistical potential (ASP)^42^ were implemented to validate the predicted binding modes and relative energies of the ligands using the GOLD v5.4 software suite. The cocrystallized ligand methotrexate was first docked, and root-mean-square deviation (RMSD) values were calculated for the heavy atoms. ASP obtained an average RMSD of 1.8757, ChemPLP of 0.8191, CS of 3.8814, and GS of 0.8149 which indicate the strong prediction power of the ChemPLP and GS scoring functions. The RMSD and docking scores are given in Tables S1 and S2, respectively.

### Cell Culture

Parent cell lines: A-431 (human epidermoid carcinoma), SW620 (human colorectal adenocarcinoma), B16–F10 (murine melanoma), and CT26.WT (murine colon carcinoma) were purchased from the American Type Culture Collection and cultured in standard cell culture conditions (37°C, 5% CO_2_, 95% relative humidity). The methotrexate-resistant sublines were obtained as previously described.^29^ The cells were grown in high-glucose Dulbecco’s Modified Eagle’s Medium (DMEM) buffered with HEPES and supplemented with Glutamax-I (Thermo Fisher Scientific Inc., Waltham, MA) with the addition of 10% v/v fetal bovine serum (EURx, Gdańsk, Poland). Care was taken to avoid cross-contamination between the cell lines (handling one cell line at a time, using antiaerosol tips and single-use equipment were the minimum safety conditions). The cells were tested every 3 months for *Mycoplasma* contamination with a MycoProbe Mycoplasma Detection Kit by R&D (Bio-Techne, Minneapolis, Minnesota).

### Proliferation Assay

The antiproliferative potential of the investigated compounds was determined using the neutral red uptake assay.^43^ Cells were washed (100 × *g*, 10 min, RT) and suspended in a custom-ordered folate-free DMEM supplemented with l-alanyl-l-glutamine and sodium pyruvate (Biowest, Nuaillé, France) with the addition of 10% v/v fetal bovine serum (FBS; EURx, Gdańsk, Poland). Then they were seeded in 96-well plates at densities of 10^4^/well (human cell lines) or 5 × 10^4^/well (murine cell lines) in a final volume of 100 μL in the folate-free complete medium and allowed to attach for 24 h. Stock solutions of synthesized compounds were freshly prepared in dimethyl sulfoxide (DMSO) and appropriately diluted in folate-free complete medium to the desired concentration. 100 μL of such solutions were added to the respective wells of the 96-well plate. The final DMSO concentration was equal in all samples, including controls, and did not exceed 0.1% v/v as it was determined to be nontoxic to the cells. After 70 h of incubation, neutral red was added to the medium to a final concentration of 1 mM. After another 2 h of incubation, the cells were washed twice with phosphate-buffered saline (PBS), destained in 200 μL extraction solution (1% acetic acid (v/v) in 50% ethanol (v/v)), and shaken for 10 min, until the dye was released from the cells. The absorbance was measured at 540 nm with an EnVision Multilabel Plate Reader (PerkinElmer, Waltham, Massachusetts). The results were calculated as the percentage of the controls and the IC_50_ values for each cell line and substance were calculated with GraphPad Prism 10.2.1 software (GraphPad Software, LLC, San Diego, California) using a four-parameter nonlinear logistic regression.

### Cell Cycle Analysis

CT26.WT were seeded in 6-well plates at a density of 10^5^ cells per well in a complete culture medium. On the next day (the time necessary for the cells to attach to the surface), the medium was replaced with folate-free DMEM supplemented with 10% FBS, and the test compounds were administered at a concentration equal to the IC_75_ value of MTX in CT26.WT cells (30 nM). After 24 or 72 h, the cells were trypsinized and fixed with ice-cold 70% v/v ethanol. Following rehydration in PBS, the cells were stained with 75 μM propidium iodide and 50 Kunitz unit/mL of RNase A in PBS for 30 min at 37°C and stored at 4°C until measurement. All samples were analyzed using a BD FACSymphony A1 cell analyzer (Becton Dickinson) at 561 nm excitation and 570/30 nm emission. Doublet discrimination and final analysis of the cellular DNA content distribution were determined using a built-in cell cycle module (Watson pragmatic algorithm) by FlowJo 7.6.1 software.

### BCECF Extrusion Assay

Following trypsinization, CT26.WT and CT26.WTM cells were suspended in the folate-free FBS-free DMEM supplemented with 100 μg/mL soybean trypsin inhibitor. The suspension was then centrifuged (100 × *g*, 10 min, RT) and the cell pellet was resuspended in folate-free DMEM to a final cell concentration of 2 × 10^6^/mL. To 750 μL of cell suspension, MTX or one of its derivatives (DMSO in a control sample) was added to a final concentration of 100 μM, and the samples were incubated at 37°C with continuous shaking at 500 rpm for 50 min. Then, 250 μL of 4 μM BCECF-AM was added to each sample, and the incubation was continued for another 10 min. The samples were then centrifuged (100 × *g*, 10 min, 4°C) and the supernatant was carefully aspirated. 750 μL of ice-cold, folate-free DMEM supplemented with 100 μM MTX or its derivatives (DMSO in the control sample) was used to resuspend the cells. Intracellular BCECF fluorescence was then immediately measured at 488 nm excitation and 530/30 nm emission (time 0) on a BD FACSymphony A1 cell analyzer (Becton Dickinson). Further measurements were performed at approximately 20-min intervals (an exact measurement time was recorded by the instrument). All samples were incubated at 37°C between measurements. Extrusion curves were plotted and the rate of dye efflux was assessed with the GraphPad Prism 10.2.1 software (GraphPad Inc.).

## Supporting Information Available

HPLC-MS analysis data; NMR spectra and molecular docking parameters (PDF)
