# Supplementary material for: Synthesis and Biological Properties of Ferrocenyl and Organic Methotrexate Derivatives
Source: ACS Omega. 2024 Jul 23;9(31):33845–56. doi: 10.1021/acsomega.4c03602 (PMC11308014; doi:10.1021/acsomega.4c03602)
Supplement: Supplementary file 1 — ao4c03602_si_001.pdf [file ao4c03602_si_001.pdf]

Synthesis and Biological Properties of Ferrocenyl and Organic Methotrexate Derivatives

Electronic Supplementary Information (ESI)

Karolina Rózga[a], Andrzej Błauż[b], Daniel Moscoh Ayine-Tora[c,d], Ernest Puścion[b], Christian G. Hartinger[c]. Damian Plażuk[a] and Błażej Rychlik*[b]*

[a] *University of Lodz, Faculty of Chemistry, Department of Organic Chemistry, 12 Tamka, 91-403 Łódź, Poland.*
[b] *University of Lodz, Faculty of Biology and Environmental Protection, Department of Oncobiology and Epigenetics, Cytometry Lab, 141/143 Pomorska, 90-236 Łódź, Poland.*
[c] *School of Chemical Sciences, University of Auckland, Private Bag 92019, Auckland 1142, New Zealand*
[d] *Department of Chemistry, University of Ghana, LG 56, Legon-Accra, Ghana*
* Corresponding authors

HPLC-MS analysis

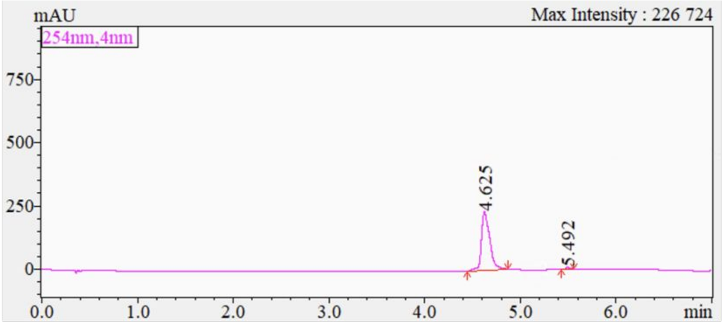

Peak#	Ret. Time	Area	Area%
1	4.625	1396774	98.429
2	5.492	22300	1.571
Total		1419073	100.000

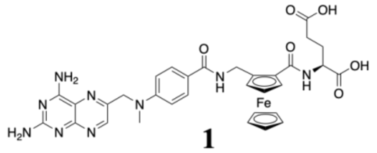

1

Chemical Formula: $C_{32}H_{33}FeN_9O_6$

m/z = 695.2

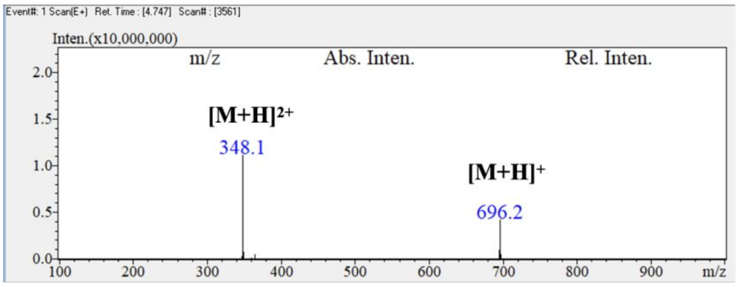

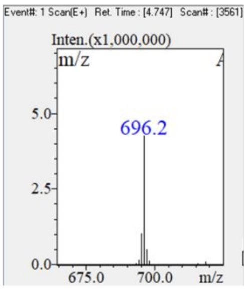

Figure S1. HPLC-MS analysis of **1**

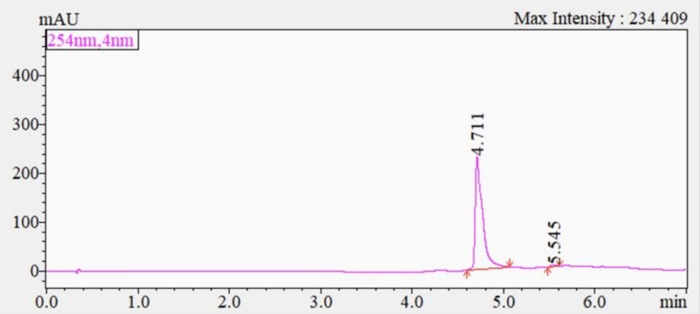

Peak#	Ret. Time	Area	Area%
1	4.711	1264300	98.779
2	5.545	15624	1.221
Total		1279924	100.000

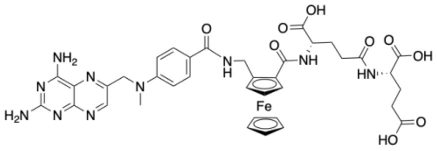

2

Chemical Formula: $C_{37}H_{40}FeN_{10}O_9$

m/z = 824.2

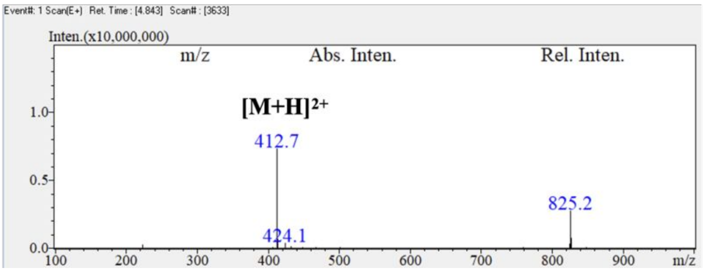

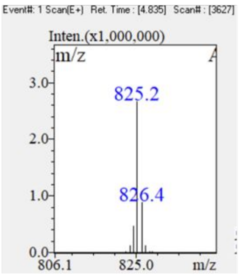

Figure S2. HPLC-MS analysis of **2**

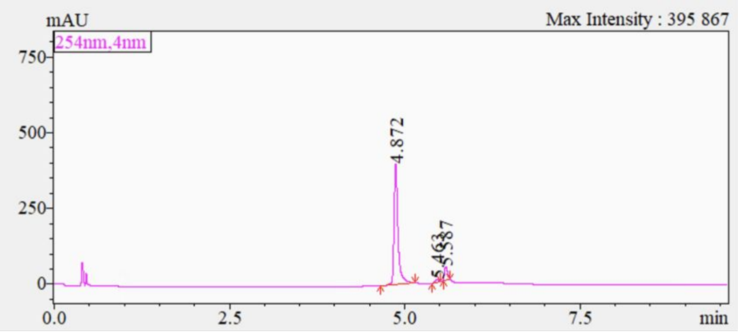

Peak#	Ret. Time	Area	Area%
1	4.872	1560008	90.148
2	5.463	33234	1.920
3	5.587	137264	7.932
Total		1730505	100.000

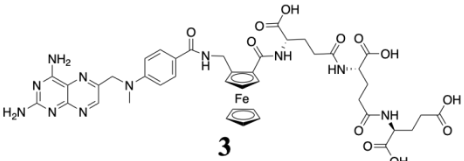

3

Chemical Formula: $C_{42}H_{47}FeN_{11}O_{12}$

m/z = 953.3

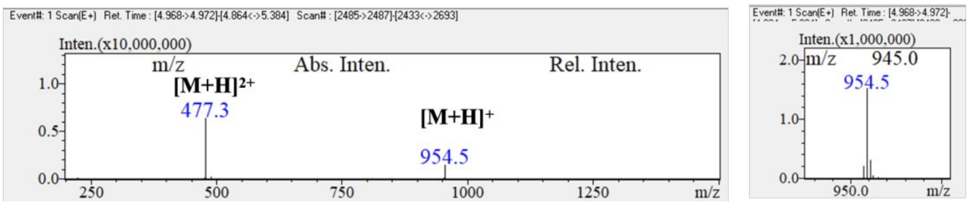

Figure S3. HPLC-MS analysis of **3**

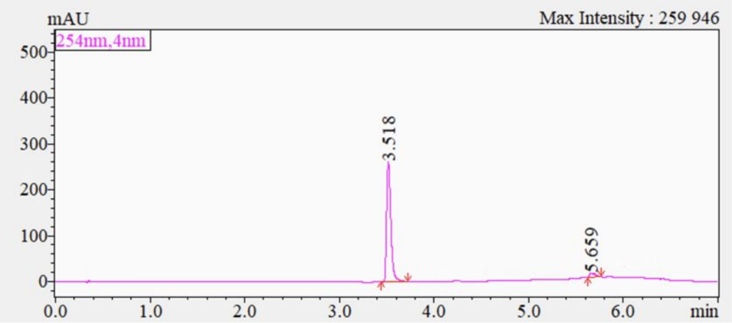

Peak#	Ret. Time	Area	Area%
1	3.518	815792	95.191
2	5.659	41211	4.809
Total		857003	100.000

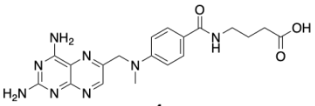

4

Chemical Formula: $C_{19}H_{22}N_8O_3$

m/z = 410.2

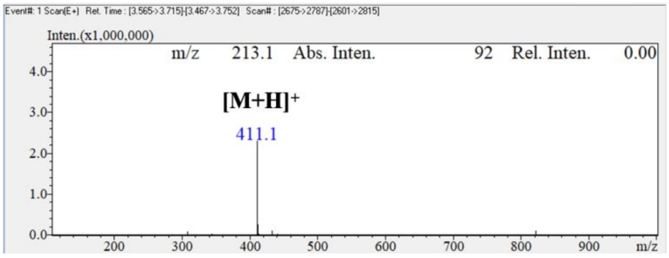

Figure S4. HPLC-MS analysis of **4**

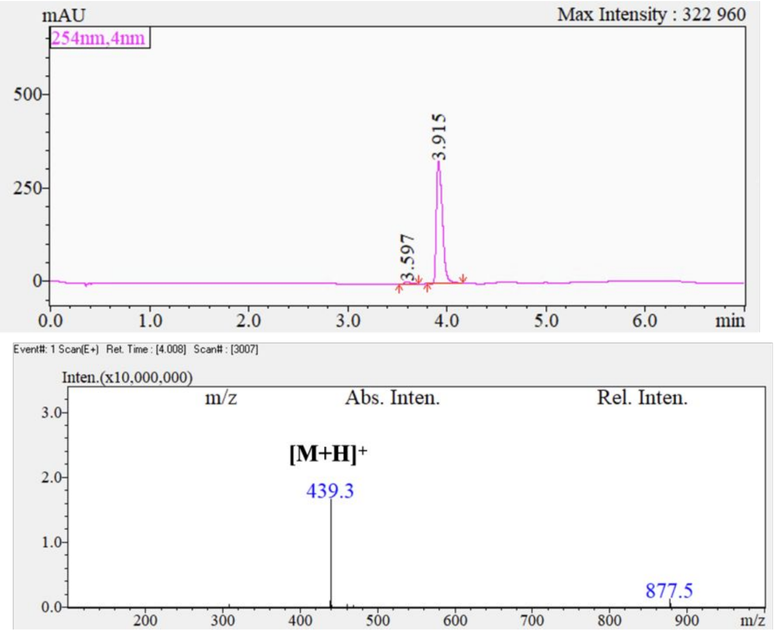

Peak#	Ret. Time	Area	Area%
1	3.597	27422	1.957
2	3.915	1373927	98.043
Total		1401350	100.000

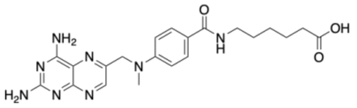

5

Chemical Formula: $C_{21}H_{26}N_8O_3$

m/z = 438.2

Figure S5. HPLC-MS analysis of **5**

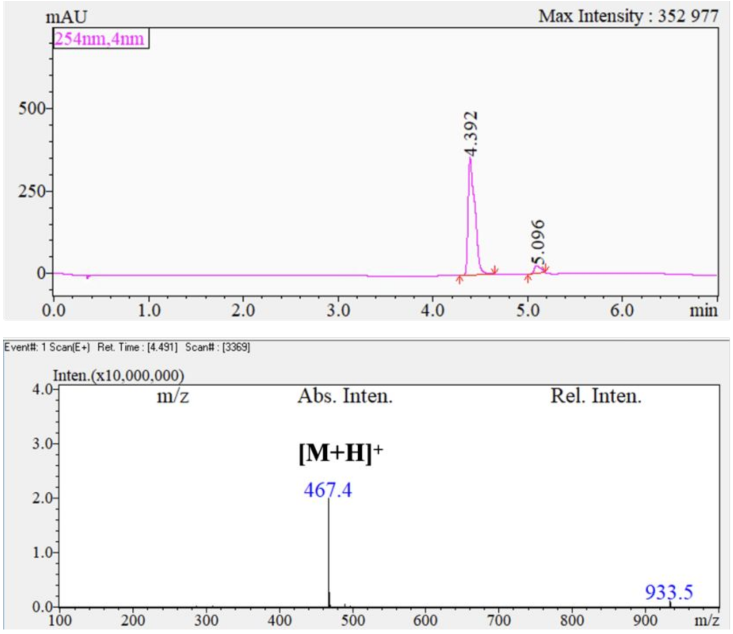

Peak#	Ret. Time	Area	Area%
1	4.392	1761774	94.736
2	5.096	97899	5.264
Total		1859673	100.000

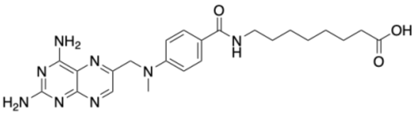

6

Chemical Formula: $C_{23}H_{30}N_8O_3$

m/z = 466.2

Figure S6. HPLC-MS analysis of **6**

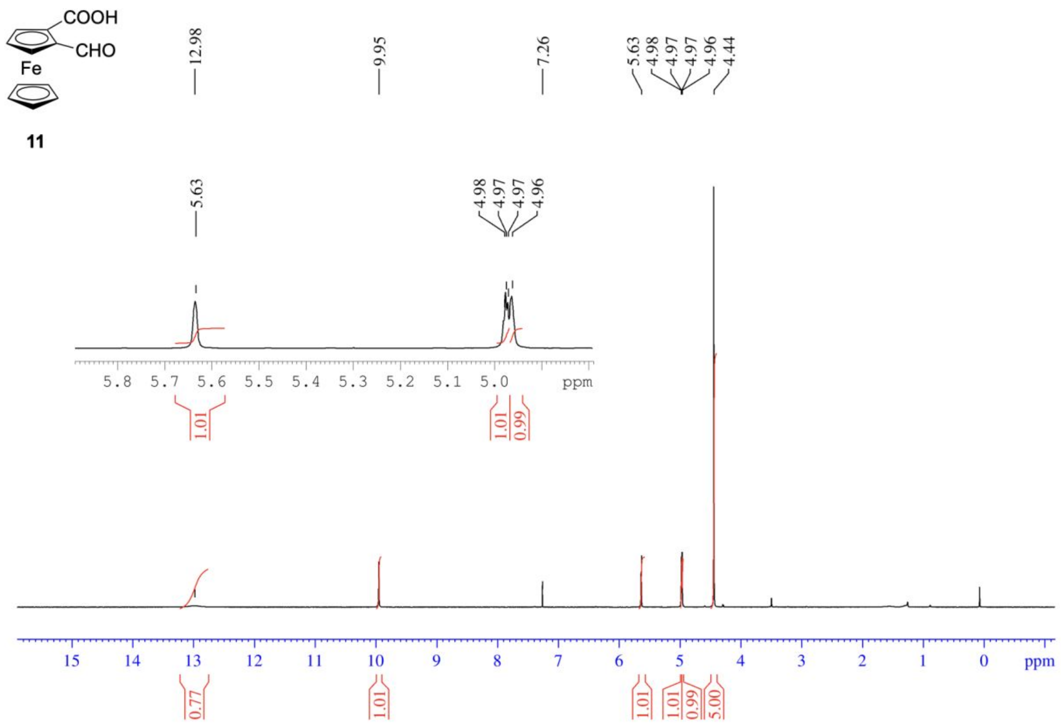

Figure S7. The ^{1}H NMR spectrum of **11** in $CDCl_3$

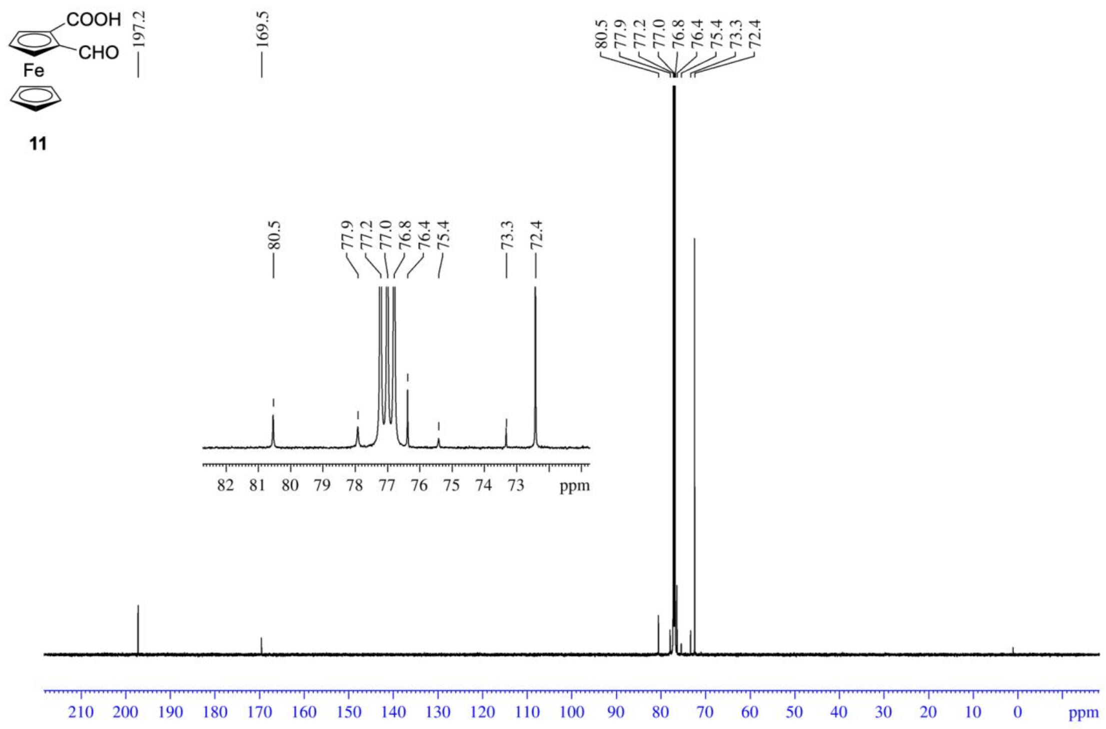

Figure S8. The $^{13}C\{^{1}H\}$ NMR spectrum of **11** in $CDCl_3$

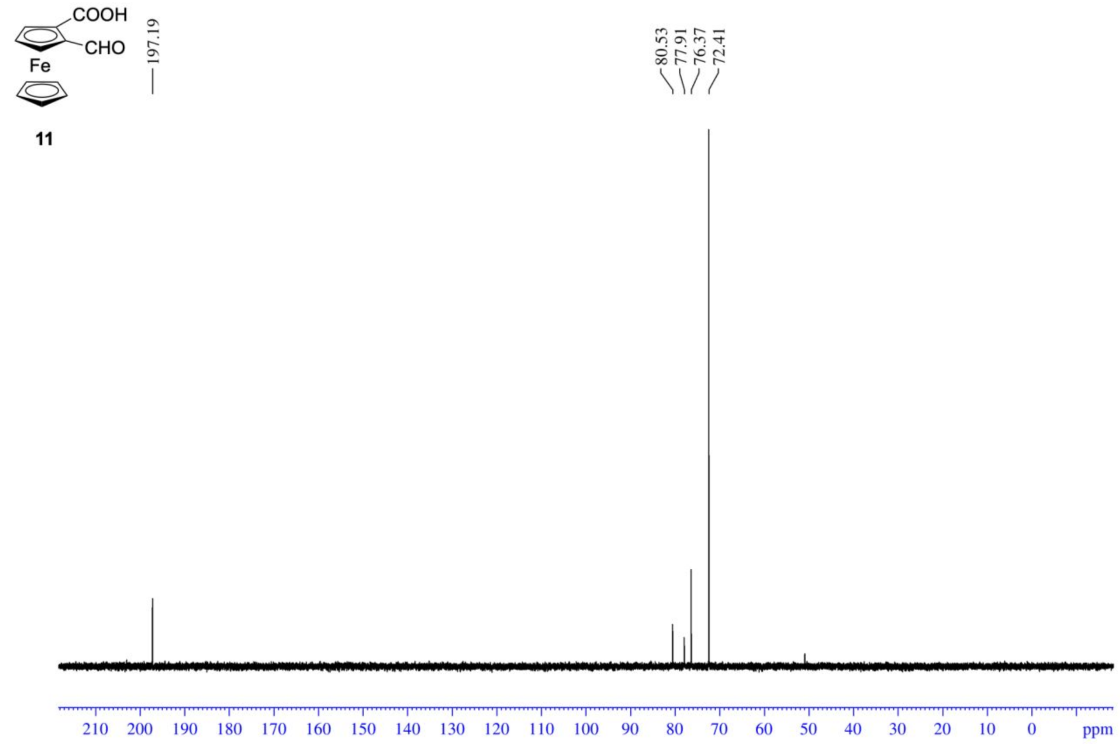

Figure S9. The DEPT 135 NMR spectrum of **11** in $CDCl_3$

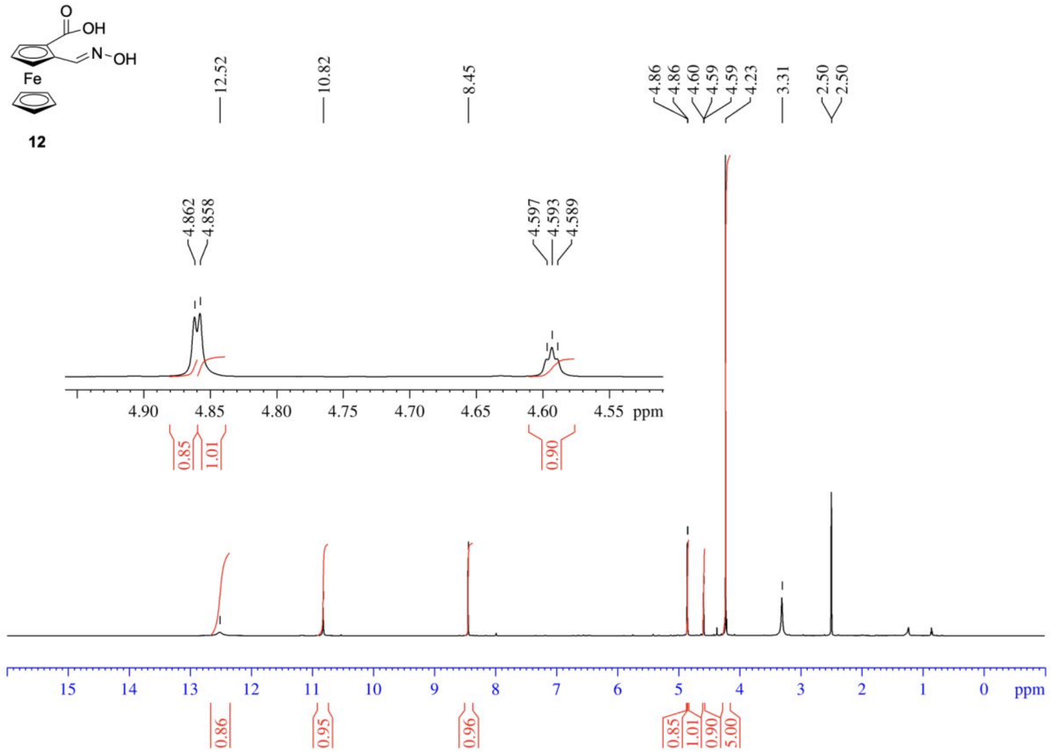

Figure S10. The ^1H NMR spectrum of **12** in DMSO-d_6

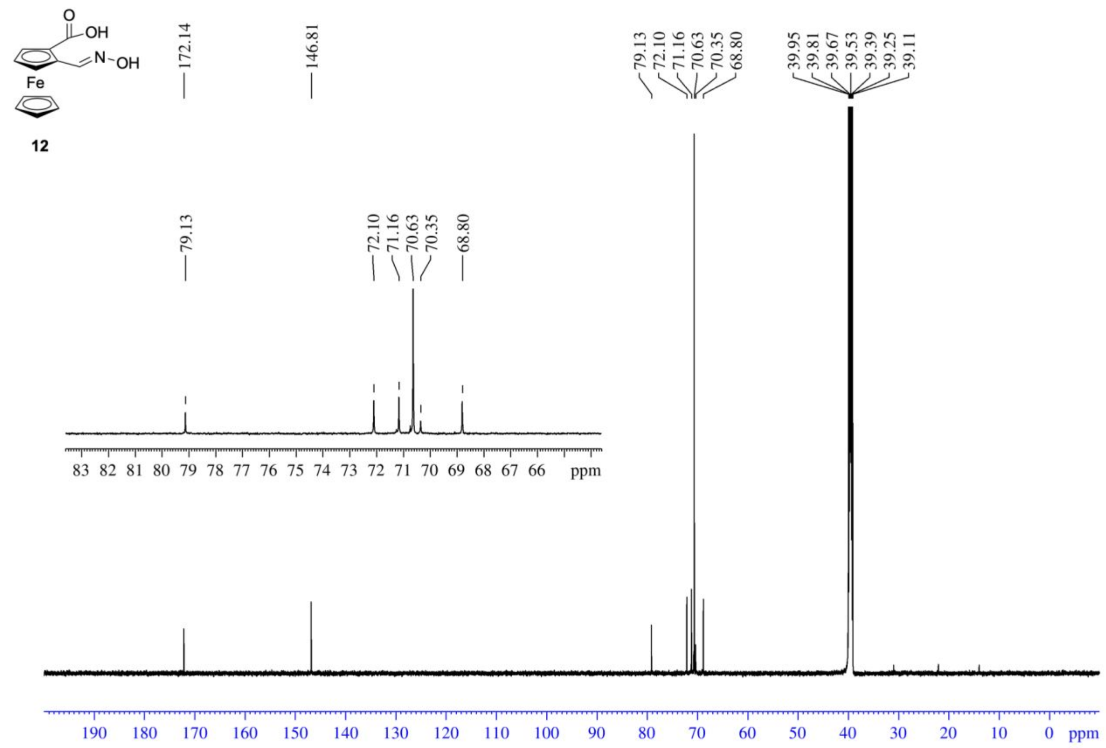

Figure S11. The $^{13}C\{^1H\}$ NMR spectrum of **12** in DMSO-d$_6$

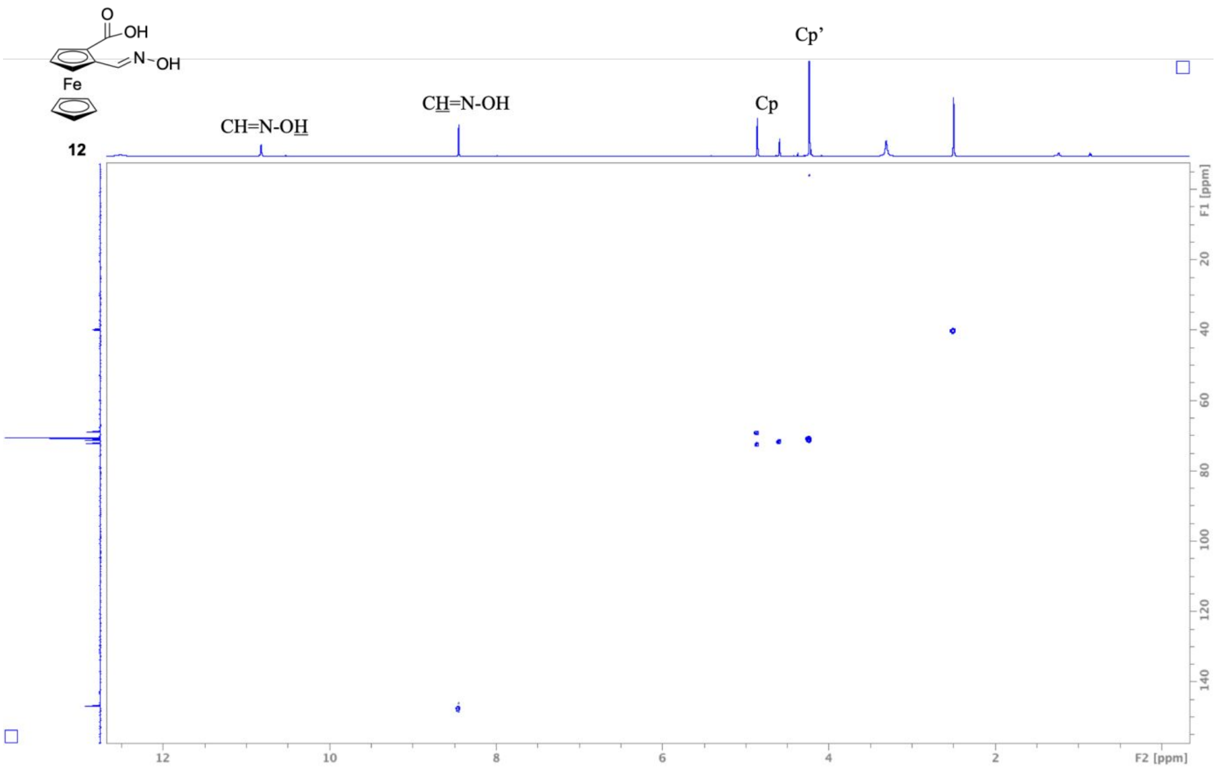

Figure S12. The ^1H-^{13}C HSQC NMR spectrum of **12** in DMSO-d$_6$

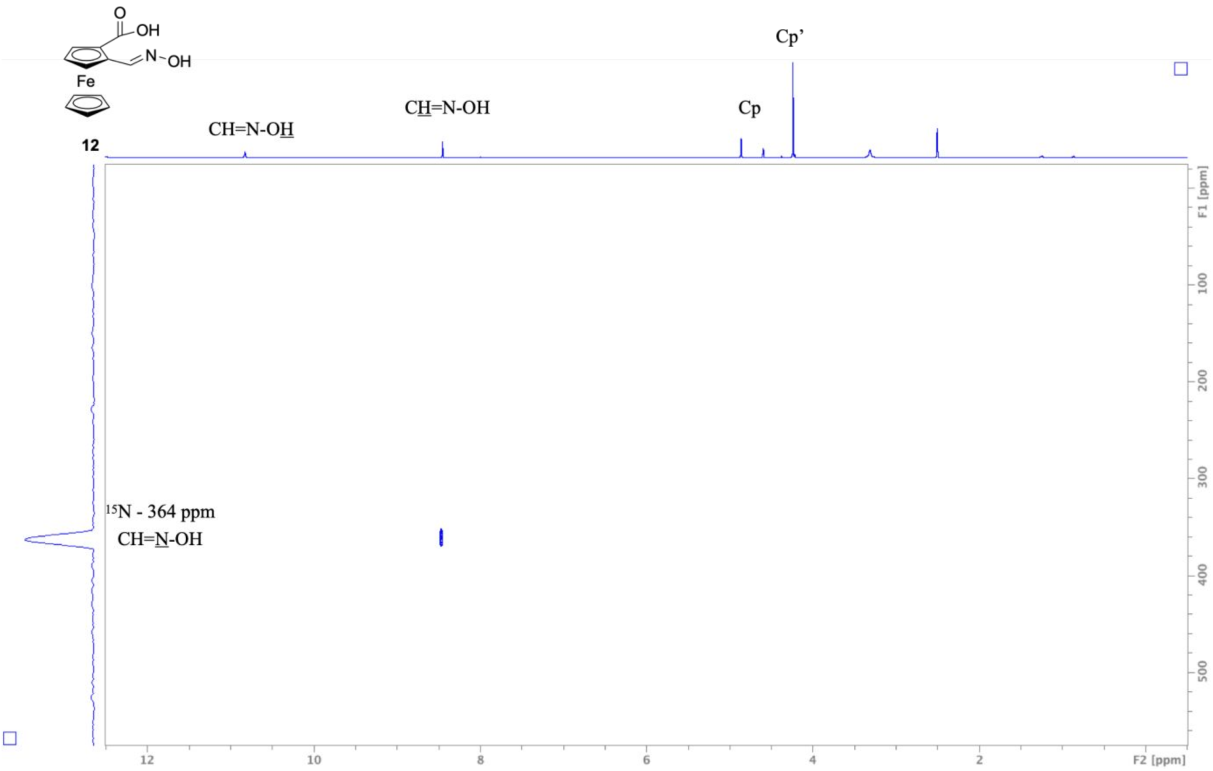

Figure S13. The ^{1}H-^{15}N HMBC NMR spectrum of **12** in DMSO-d_6

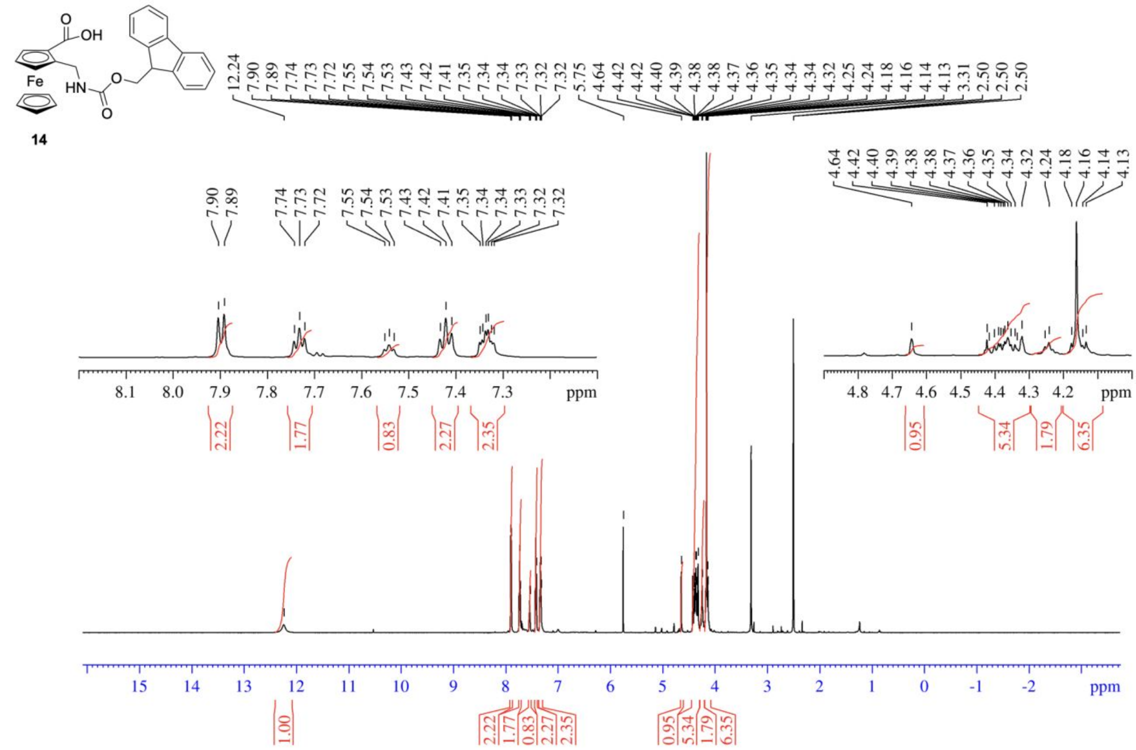

Figure S14. The ^{1}H NMR spectrum of **14** in DMSO-d_6

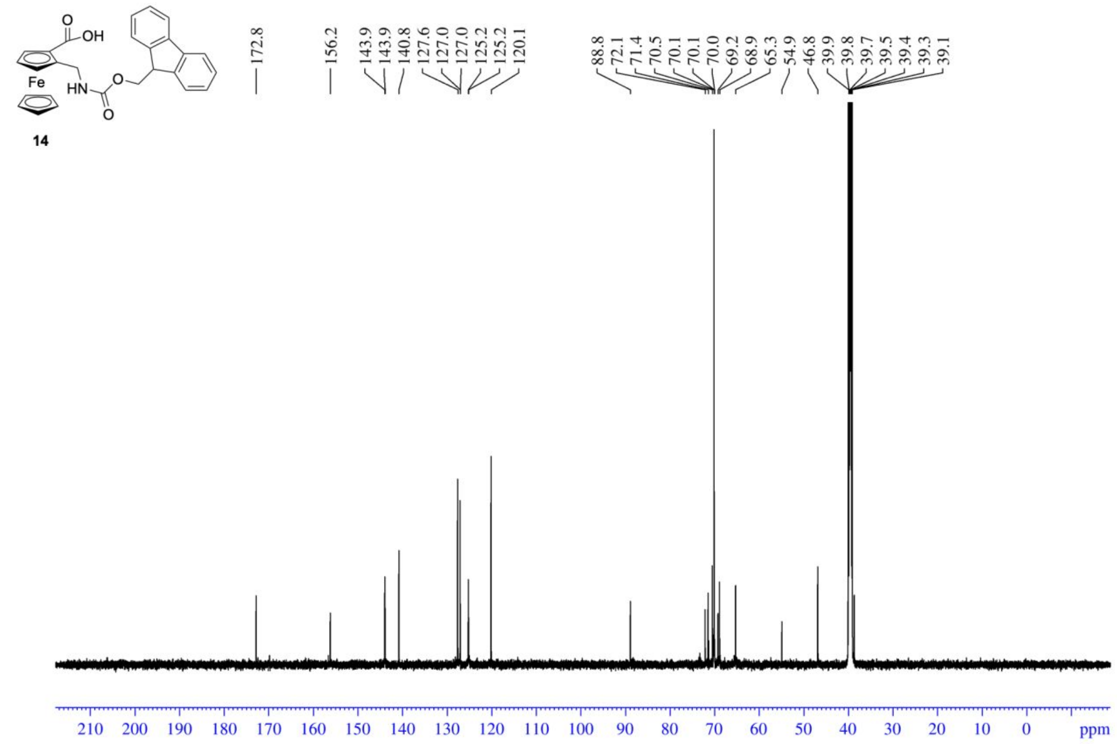

Figure S15. The $^{13}C\{^{1}H\}$ NMR spectrum of **14** in DMSO-d$_6$

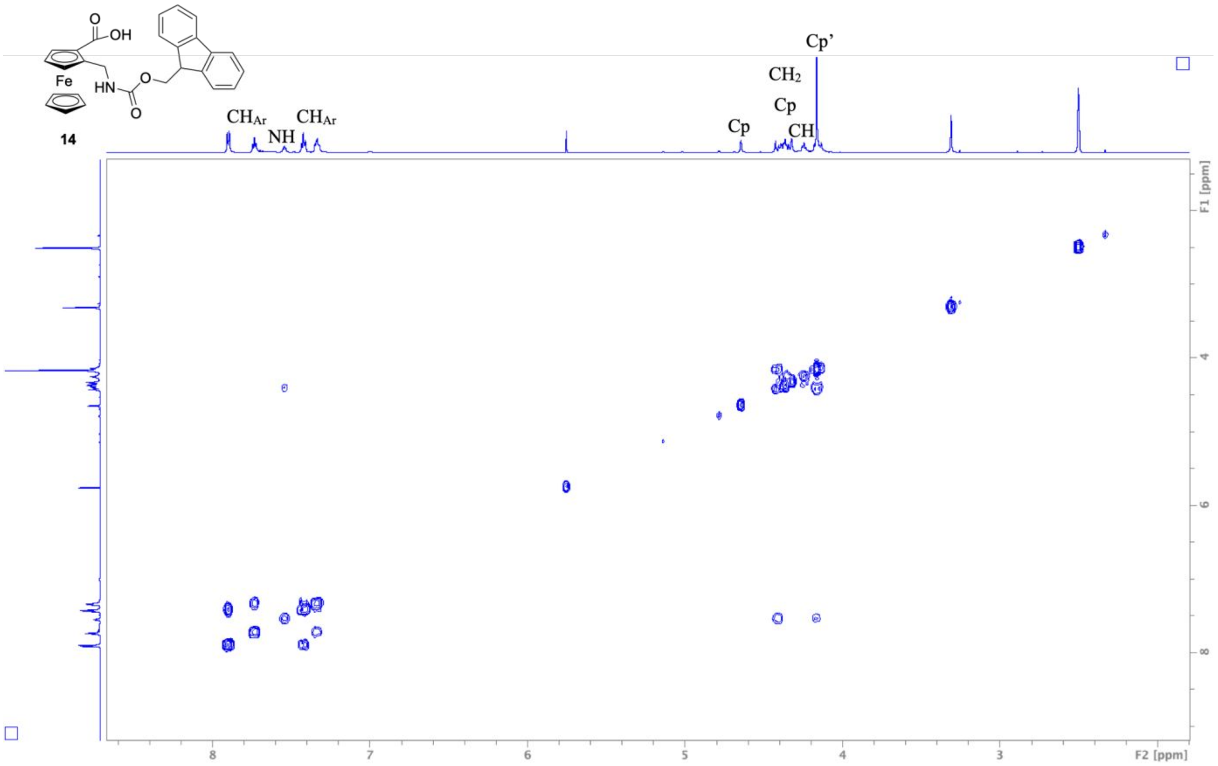

Figure S16. The ^{1}H-^{1}H COSY NMR spectrum of **14** in DMSO-d$_6$

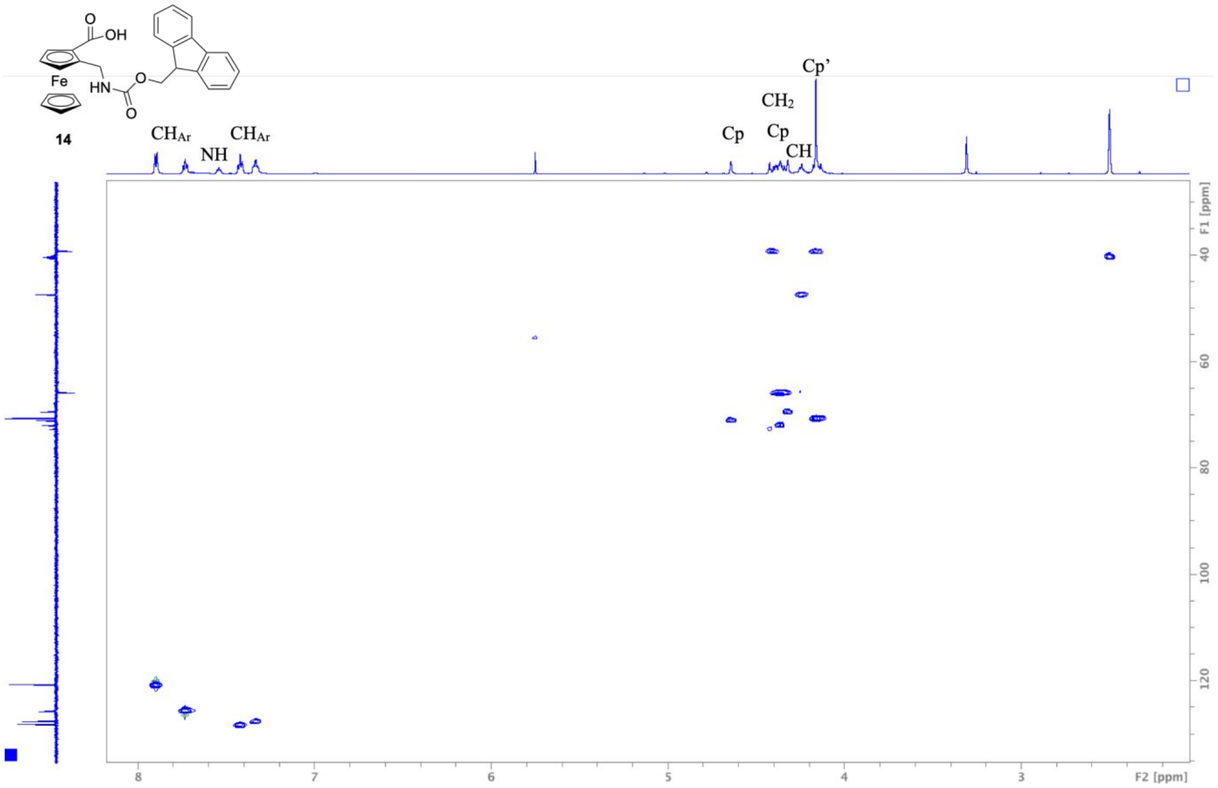

Figure S17. The ^{1}H-^{13}C HSQC NMR spectrum of **14** in DMSO-d_6

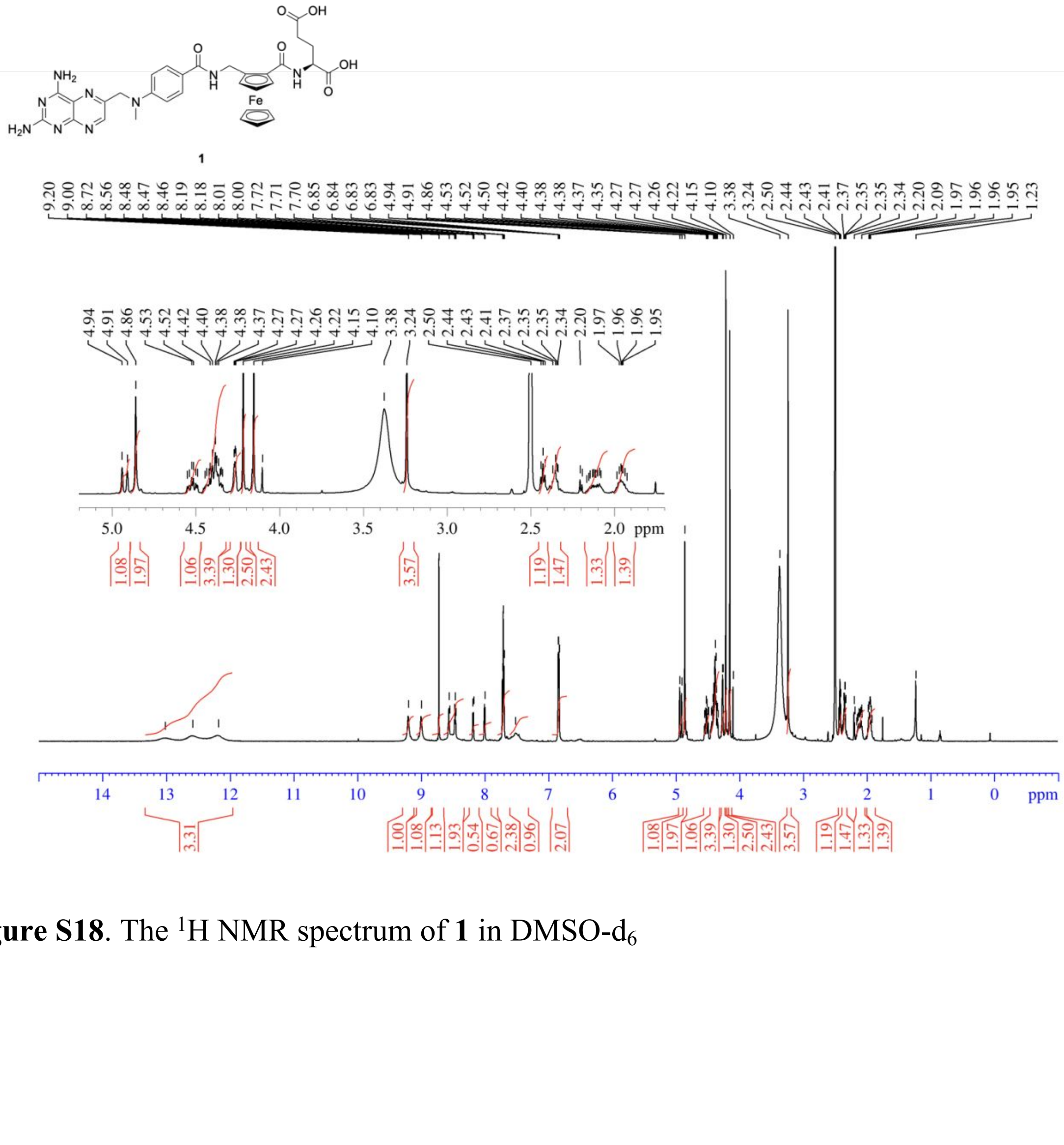

Figure S18. The ^{1}H NMR spectrum of **1** in DMSO-d_6

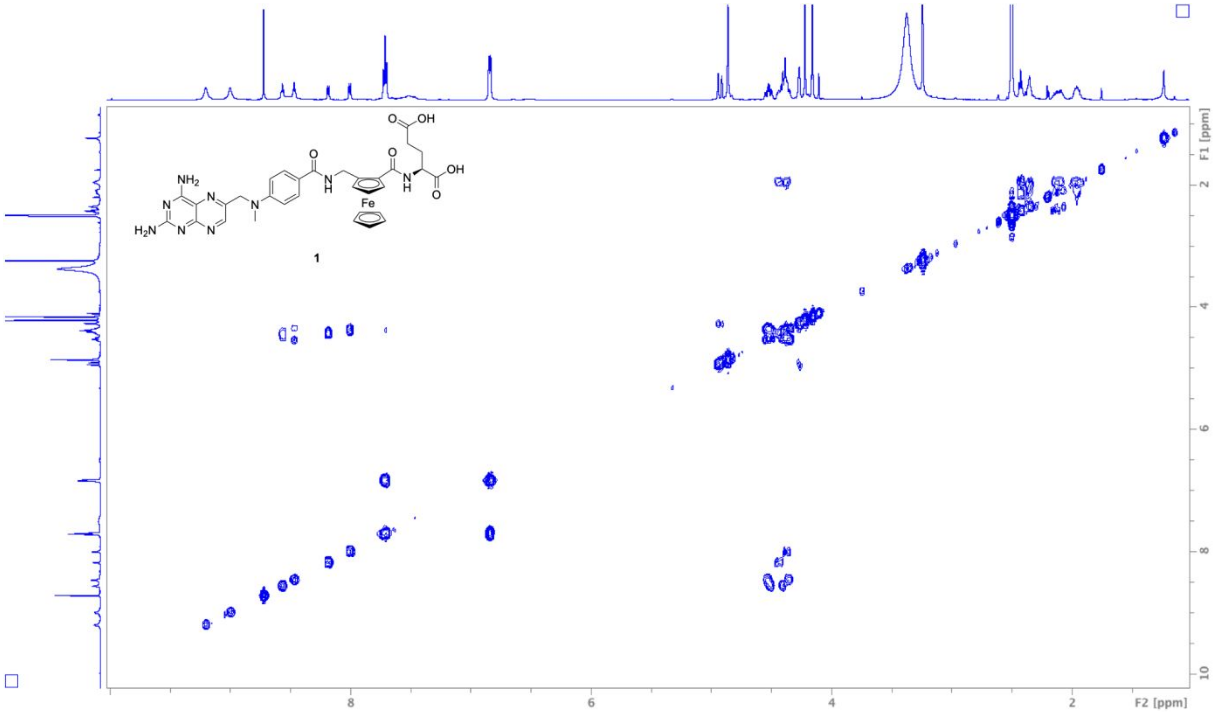

Figure S19. The ^1H-^1H COSY NMR spectrum of **1** in DMSO-d_6

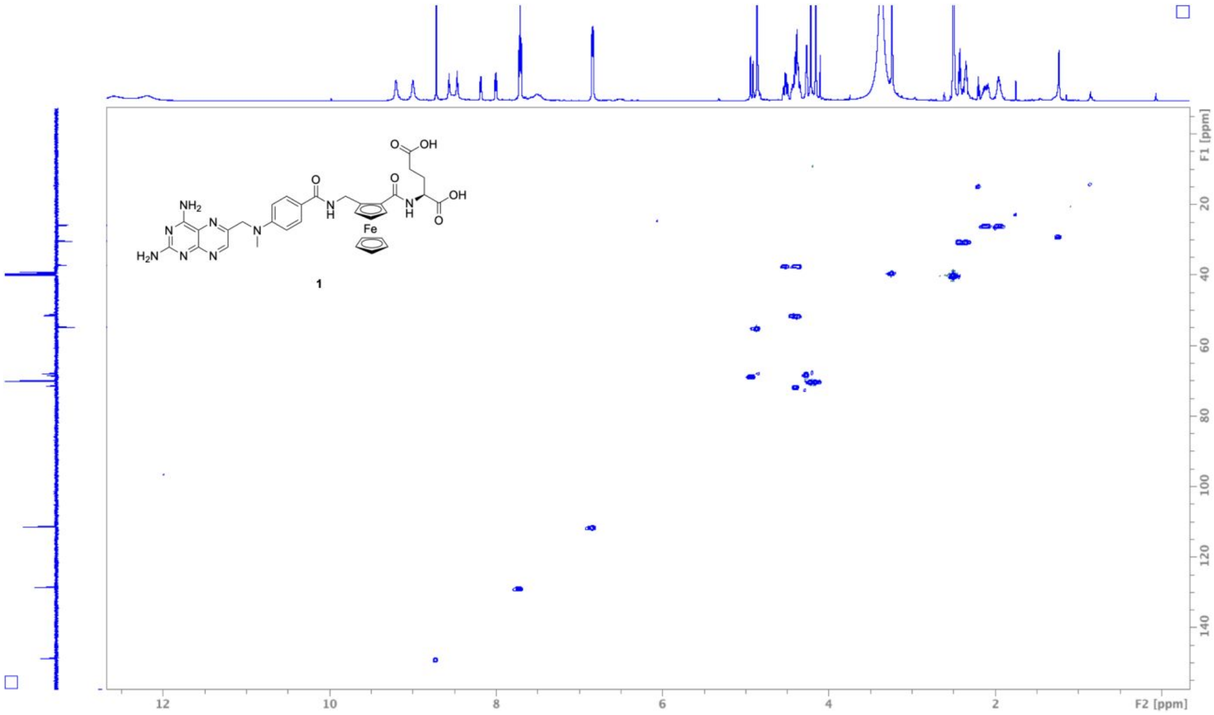

Figure S20. The ^1H-^{13}C HSQC NMR spectrum of **1** in DMSO-d_6

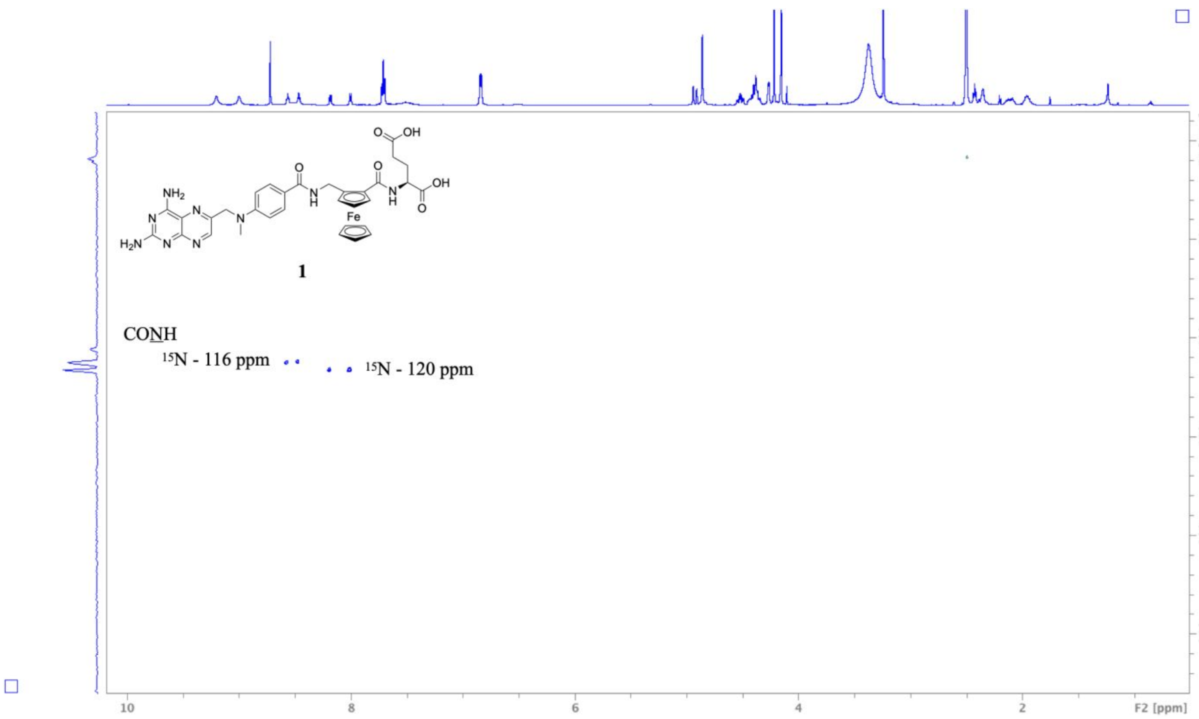

Figure S21. The ^{1}H-^{15}N HSQC NMR spectrum of **1** in DMSO-d_6

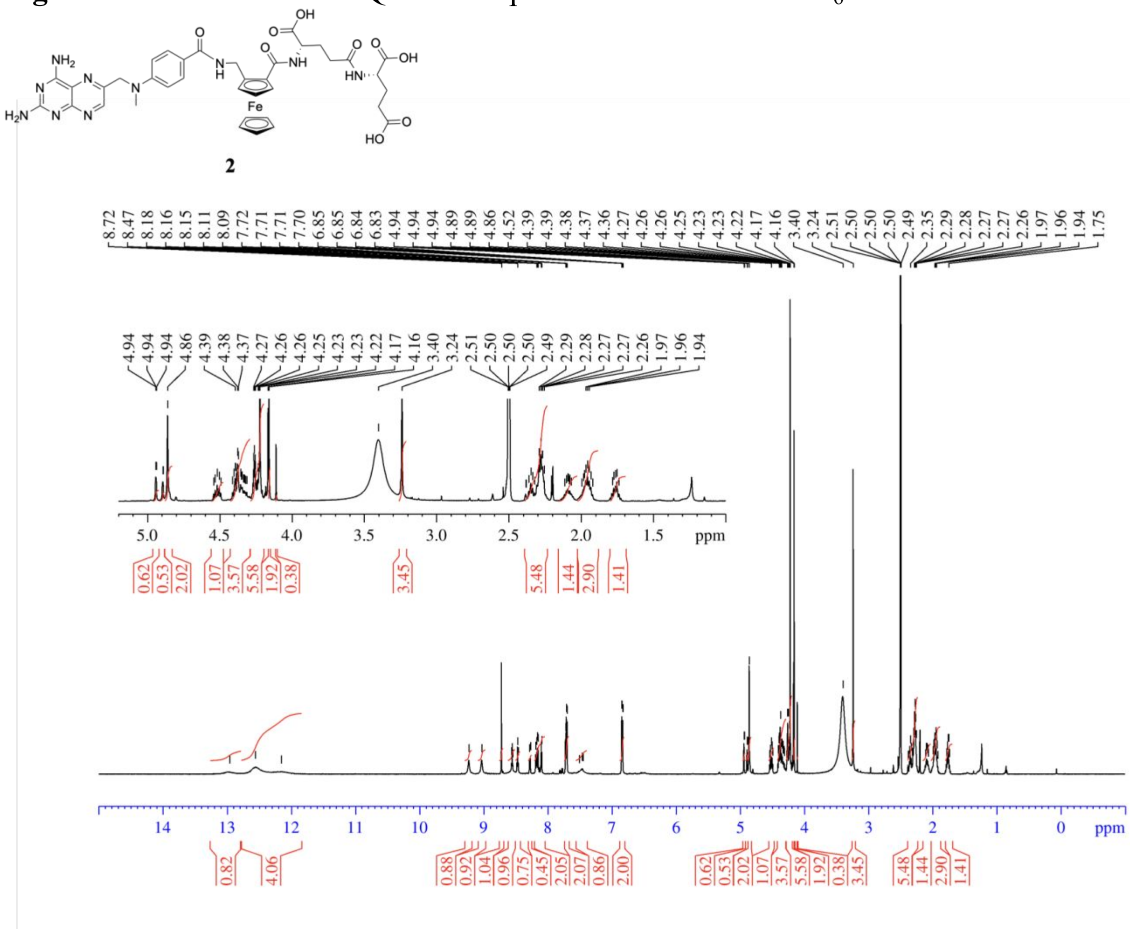

Figure S22. The ^{1}H NMR spectrum of **2** in DMSO-d_6

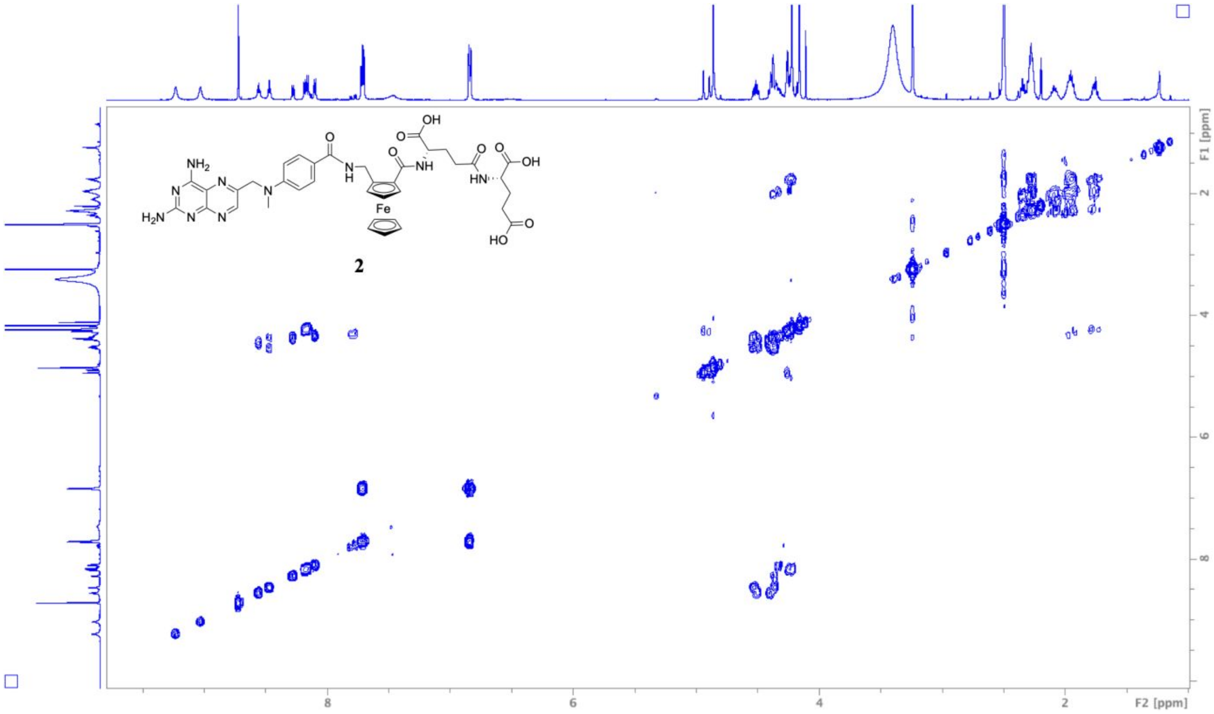

Figure S23. The ^{1}H-^{1}H COSY NMR spectrum of **2** in DMSO-d_6

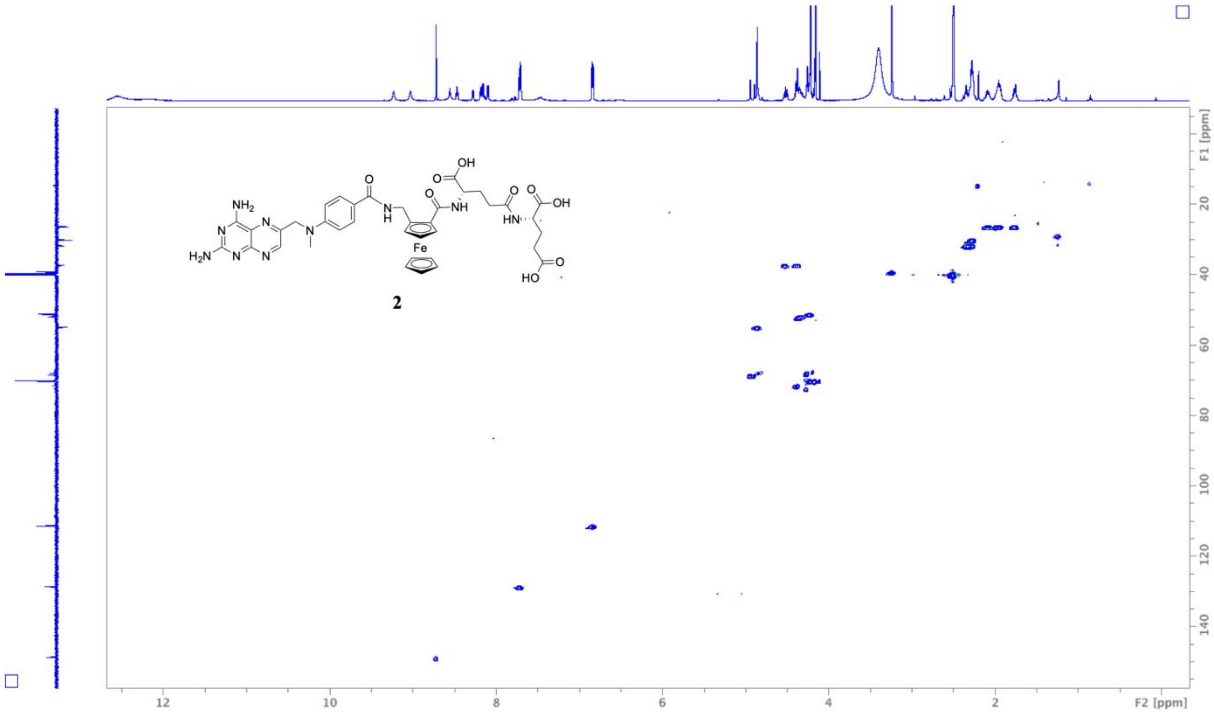

Figure S24. The ^{1}H-^{13}C HSQC NMR spectrum of **2** in DMSO-d_6

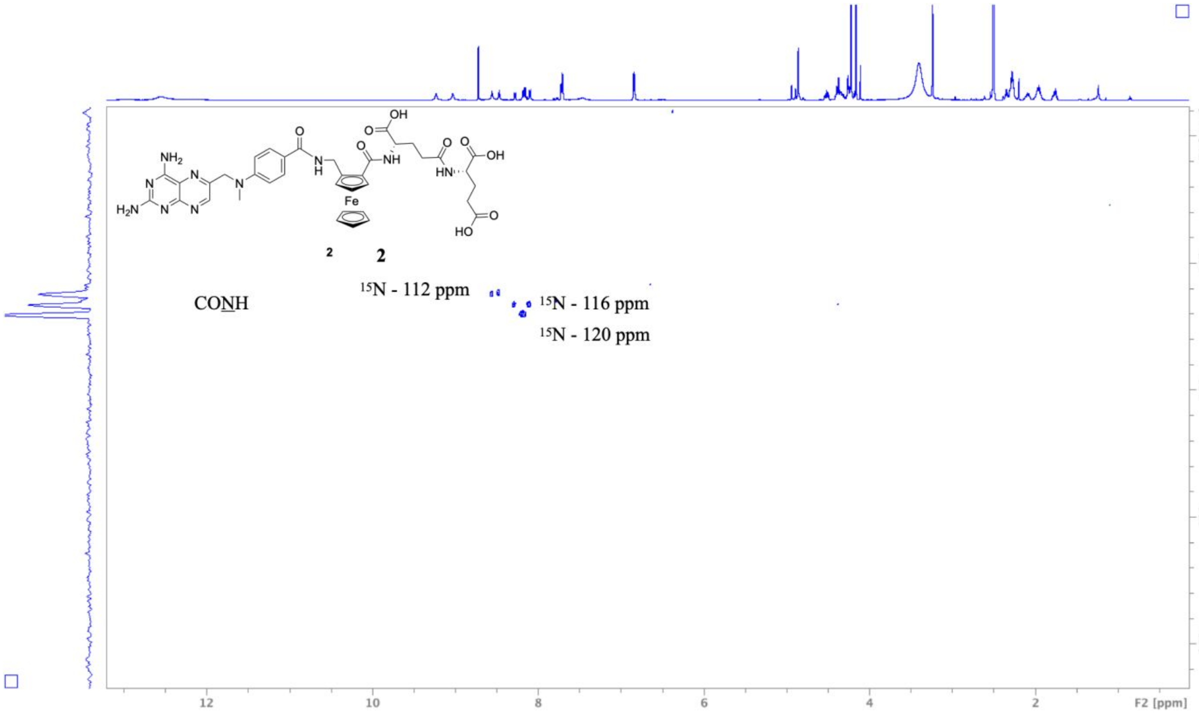

Figure S25. The ^{1}H-^{15}N HSQC NMR spectrum of **2** in DMSO-d_6

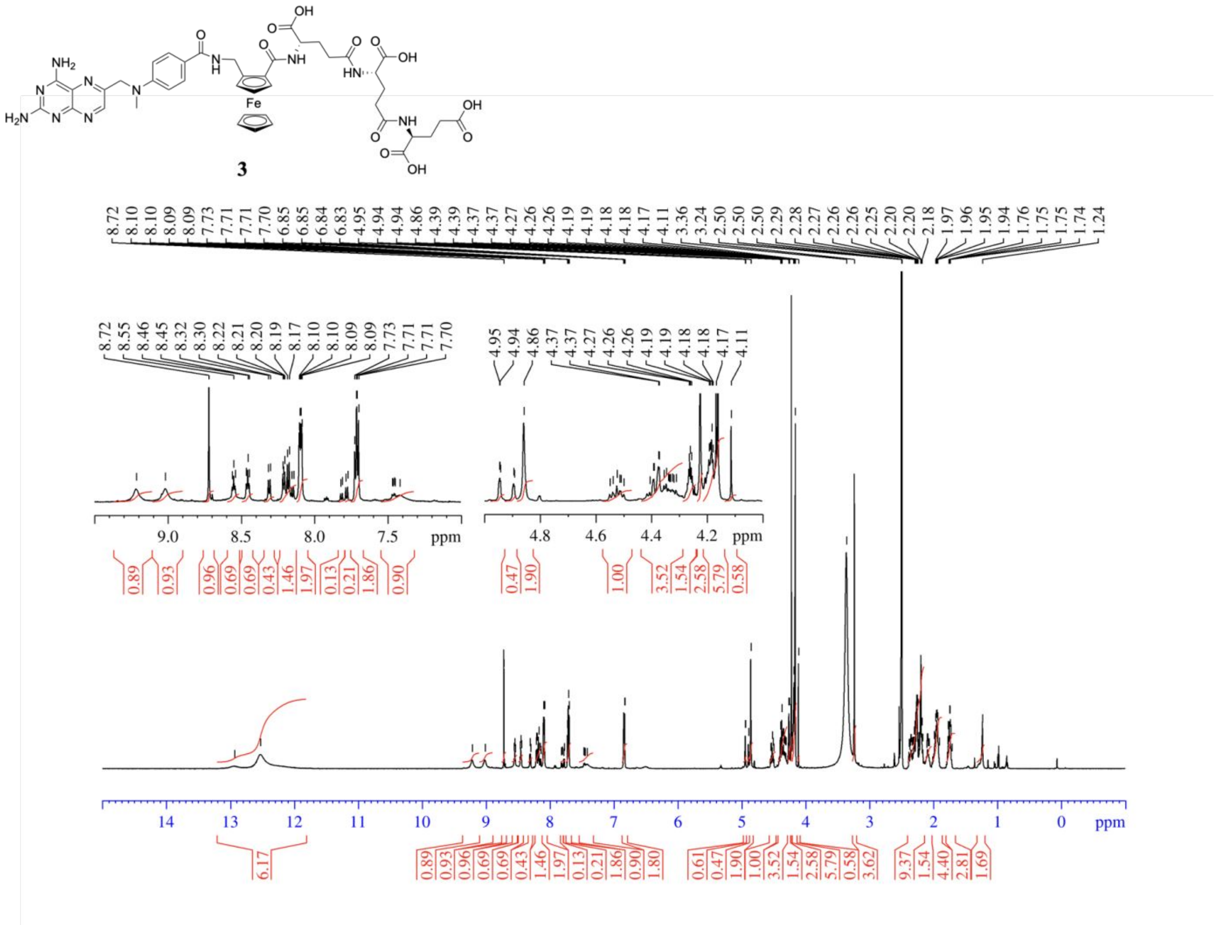

Figure S26. The ^{1}H NMR spectrum of **3** in DMSO-d_6

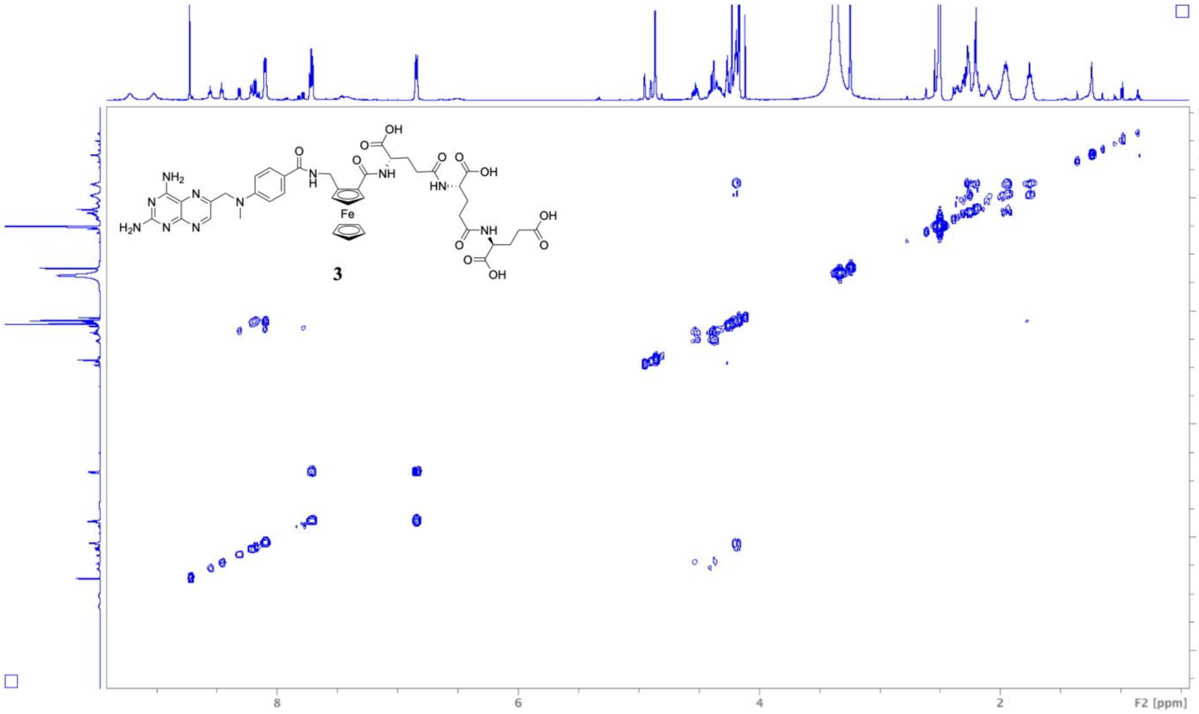

Figure S27. The ^{1}H-^{1}H COSY NMR spectrum of **3** in DMSO-d_6

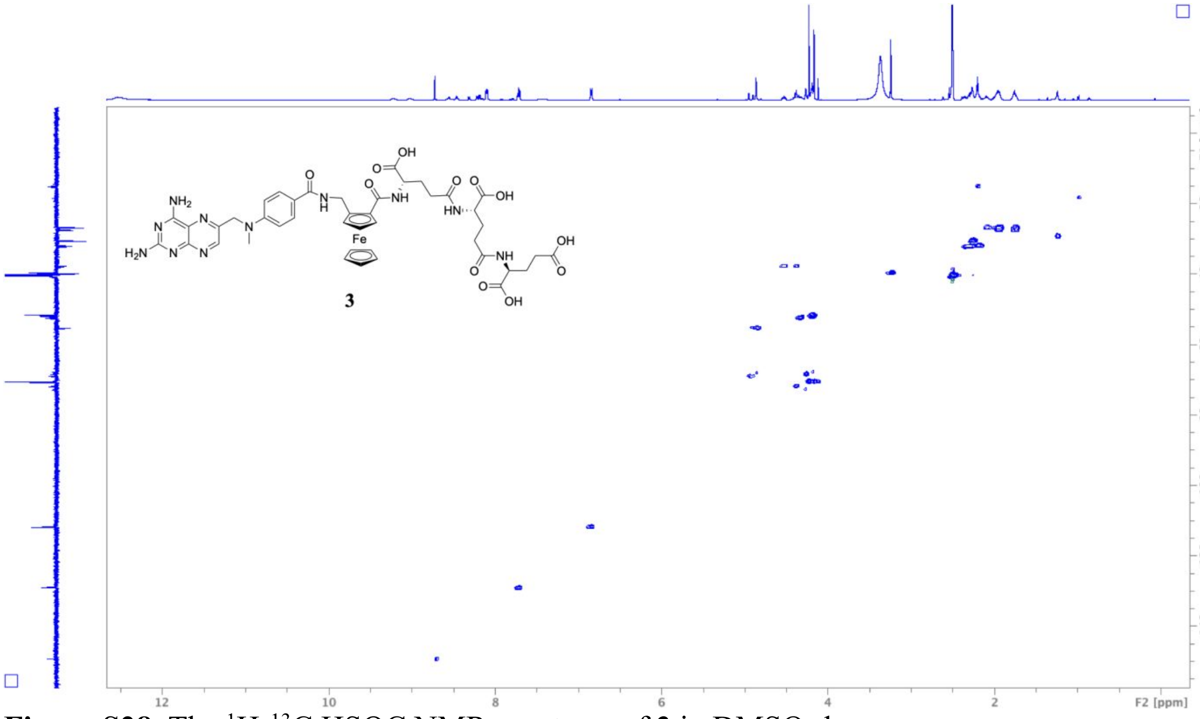

Figure S28. The ^{1}H-^{13}C HSQC NMR spectrum of **3** in DMSO-d_6

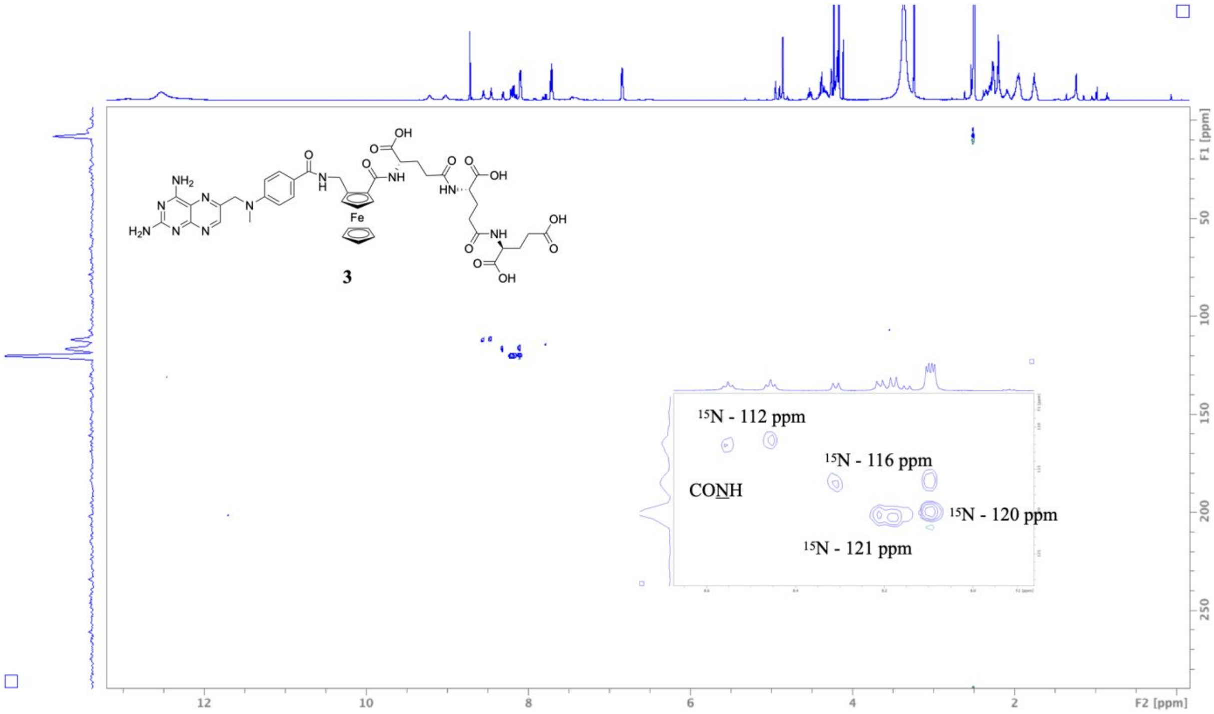

Figure S29. The ^{1}H-^{15}N HSQC NMR spectrum of **3** in DMSO-d_6

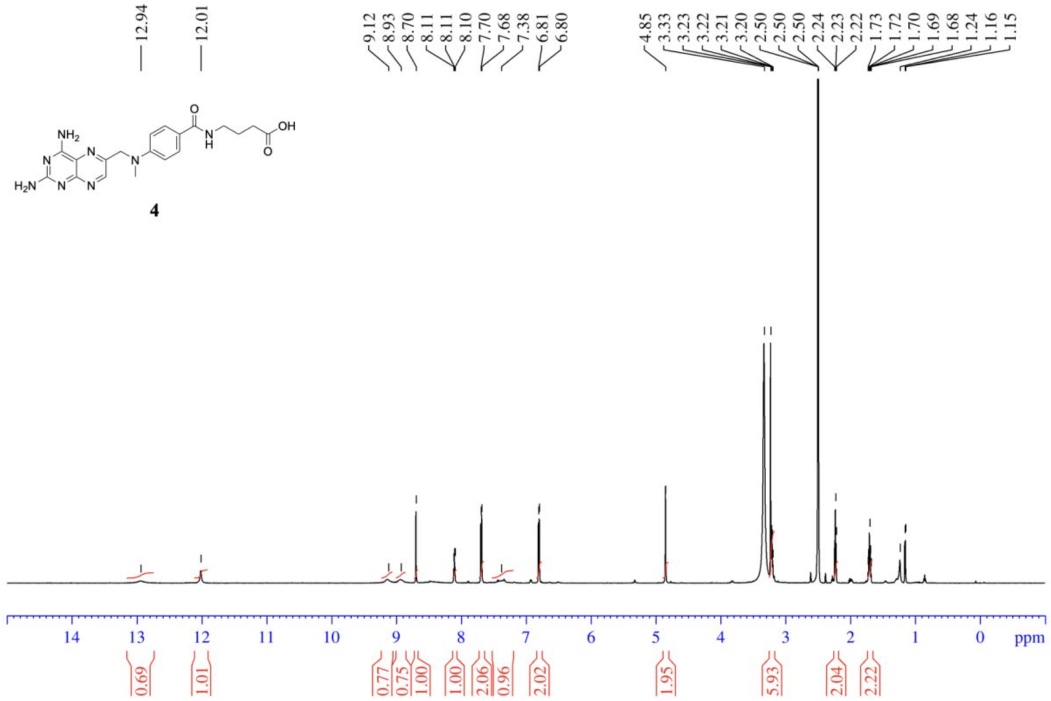

Figure S30. The ^{1}H NMR spectrum of **4** in DMSO-d_6

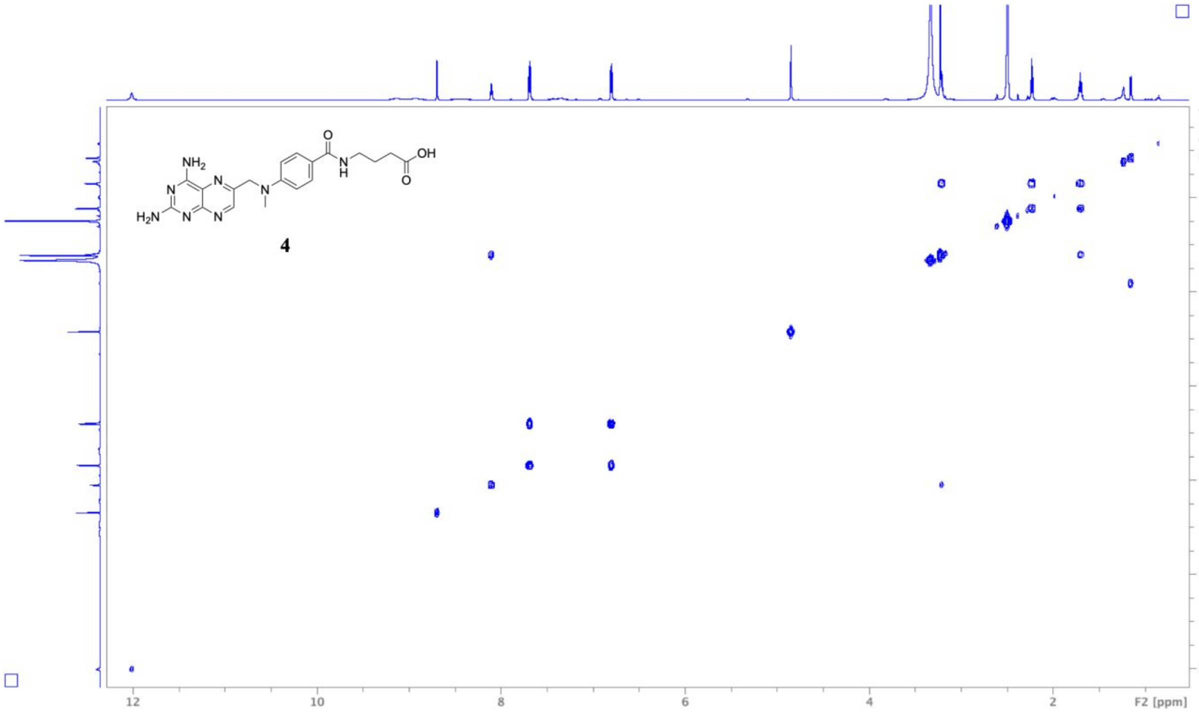

Figure S31. The ^{1}H-^{1}H COSY NMR spectrum of **4** in DMSO-d_6

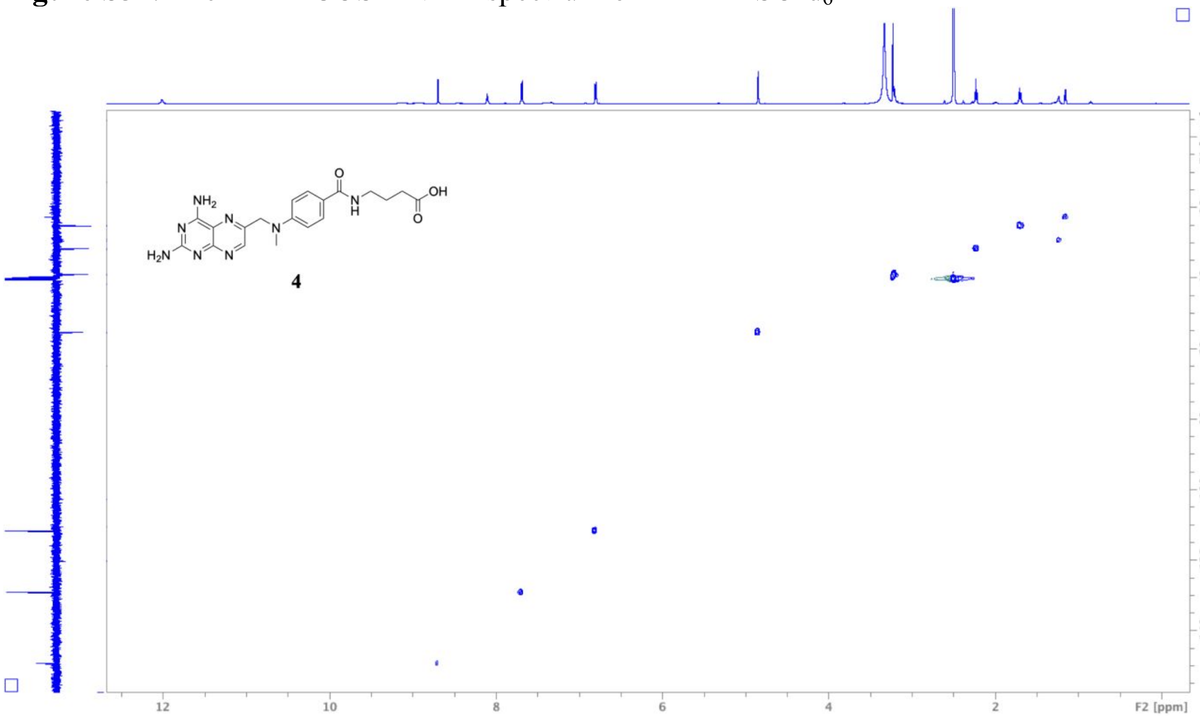

Figure S32. The ^{1}H-^{13}C HSQC NMR spectrum of **4** in DMSO-d_6

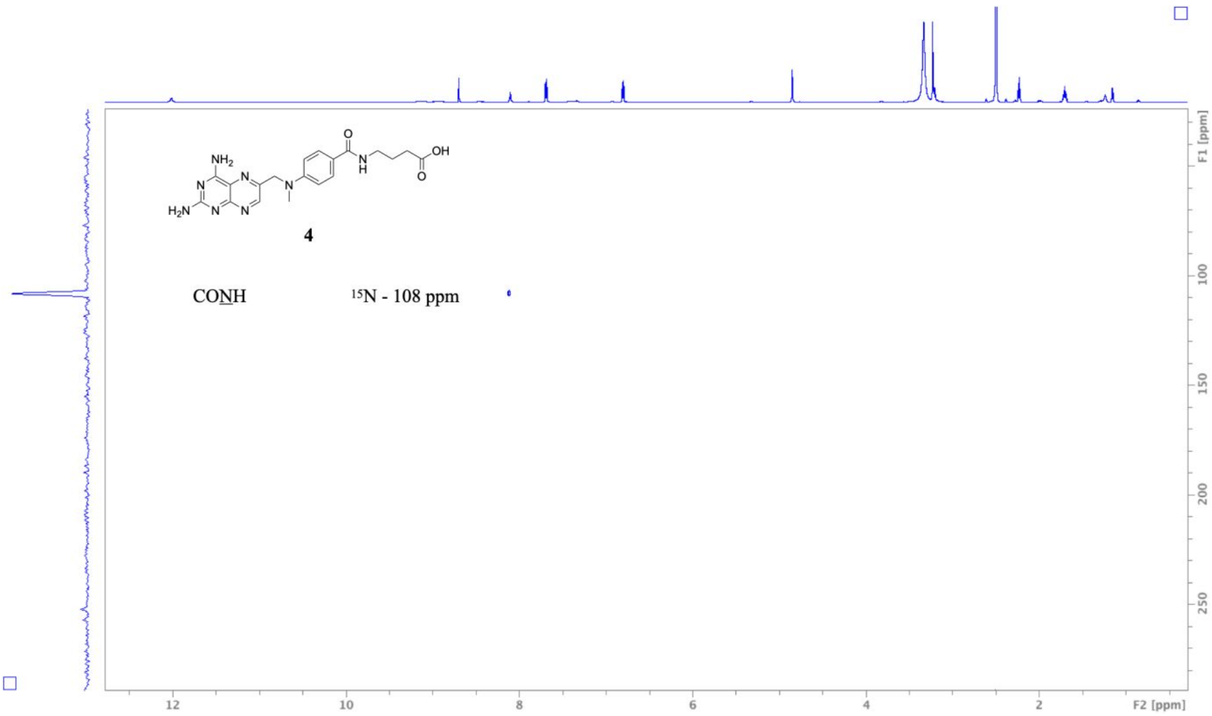

Figure S33. The ^{1}H-^{15}N HSQC NMR spectrum of **2** in DMSO-d_6

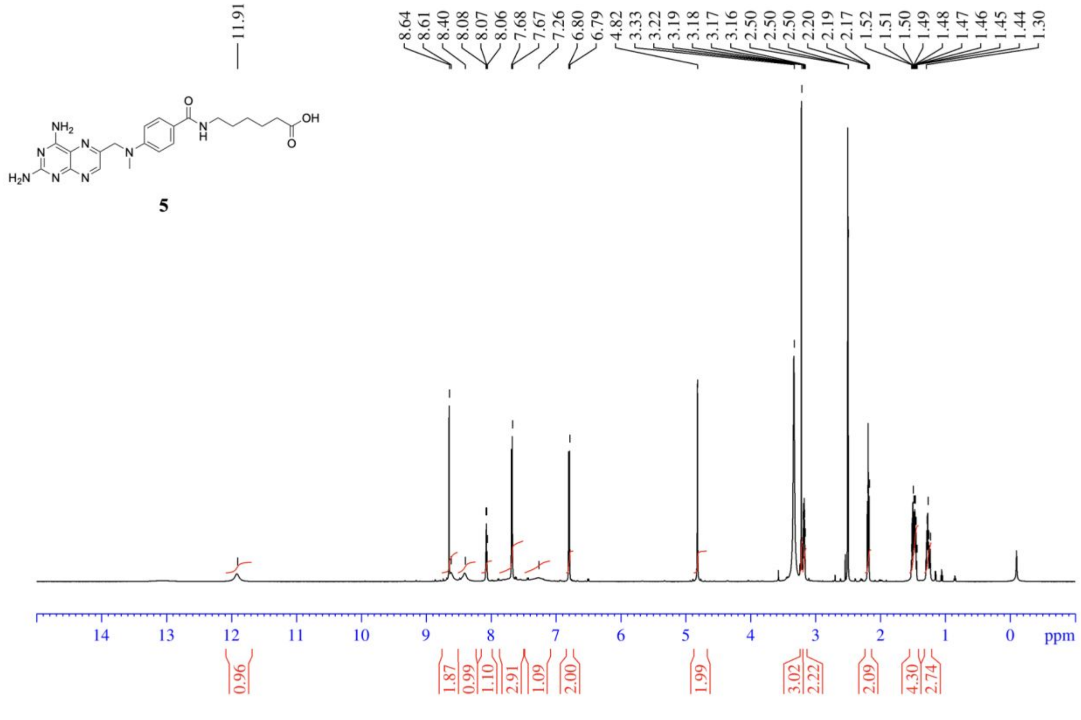

Figure S34. The ^{1}H NMR spectrum of **5** in DMSO-d_6

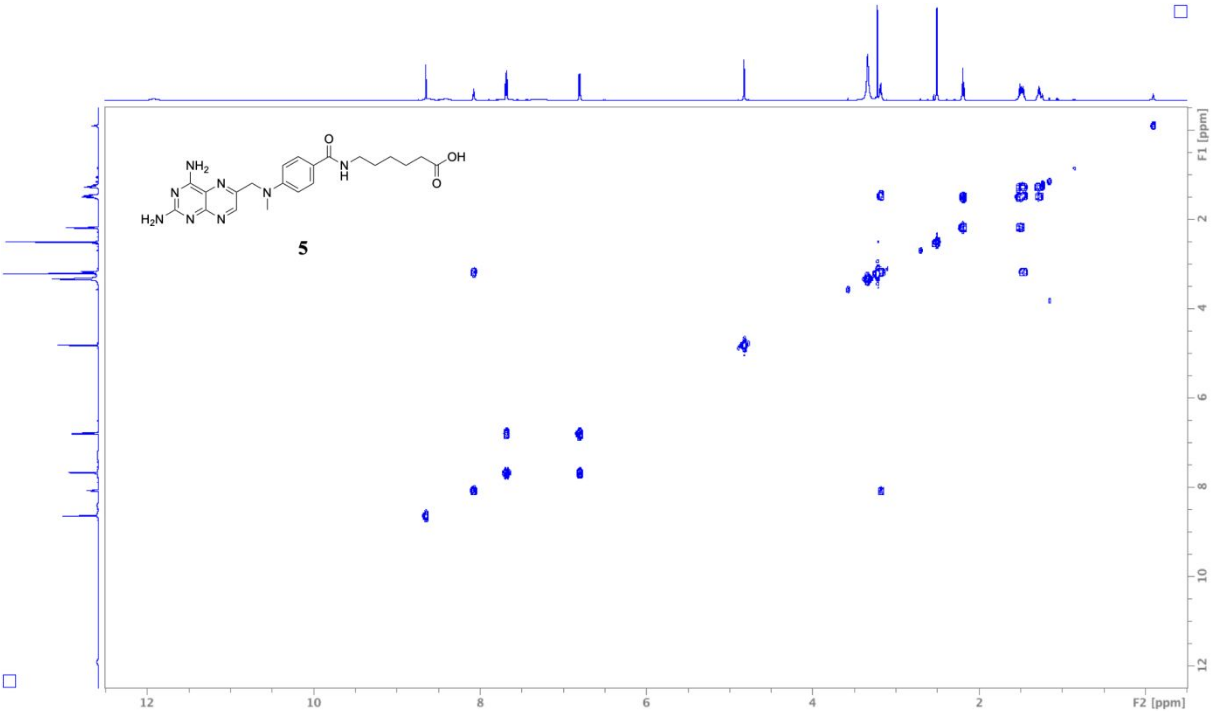

Figure S35. The ^{1}H-^{1}H COSY NMR spectrum of **5** in DMSO-d$_6$

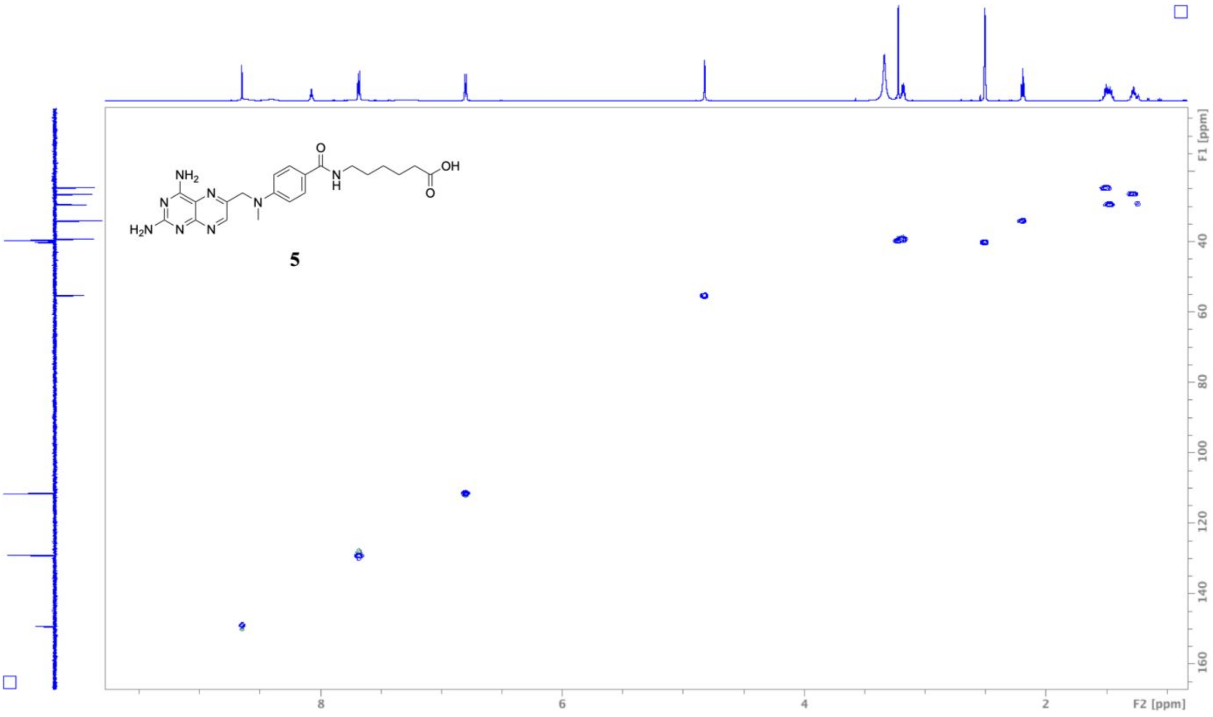

Figure S36. The ^{1}H-^{13}C HSQC NMR spectrum of **5** in DMSO-d$_6$

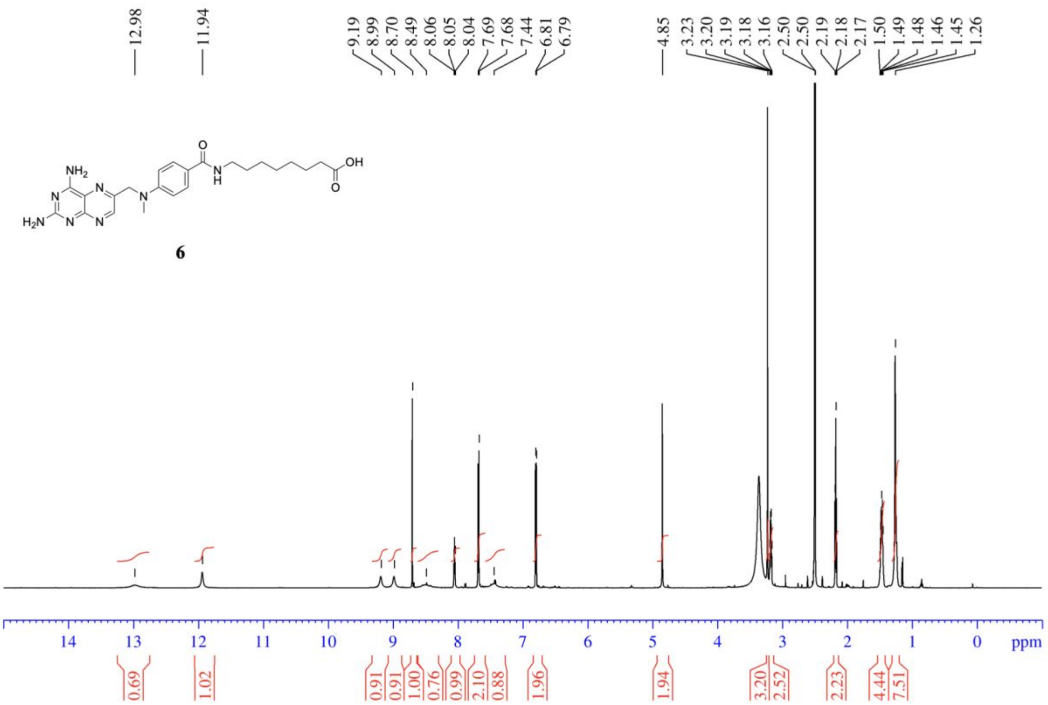

Figure S37. The ^{1}H NMR spectrum of **6** in DMSO-d_6

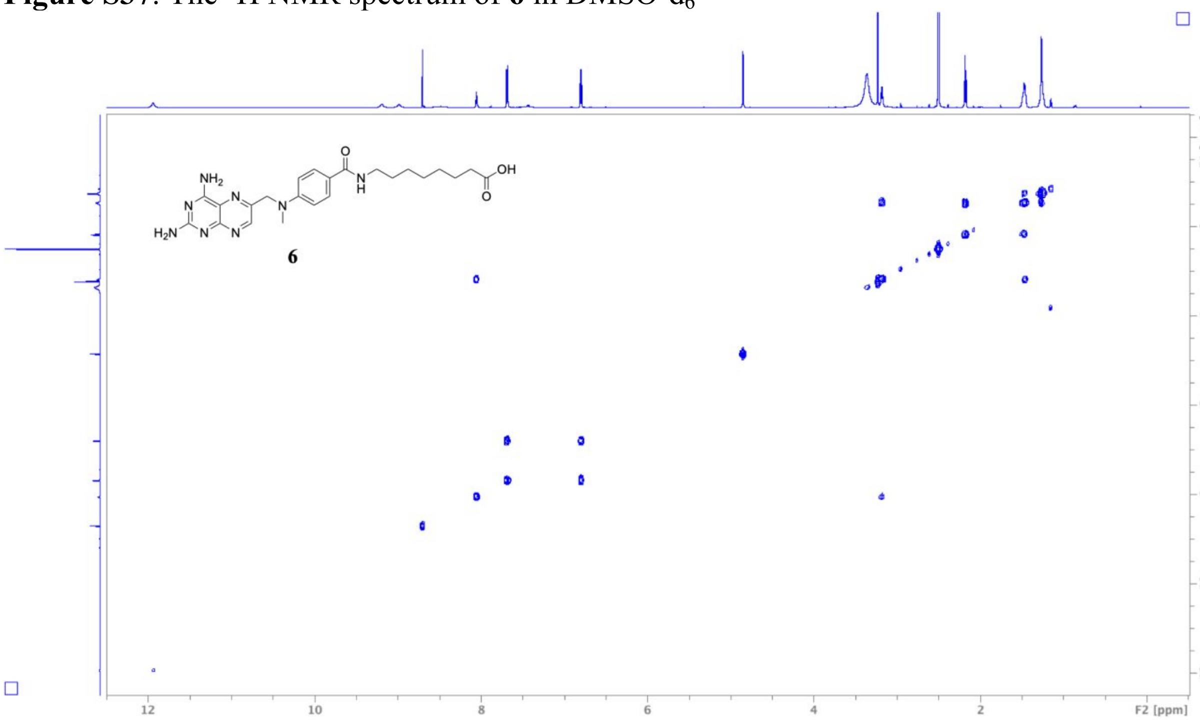

Figure S38. The ^{1}H-^{1}H COSY NMR spectrum of **6** in DMSO-d_6

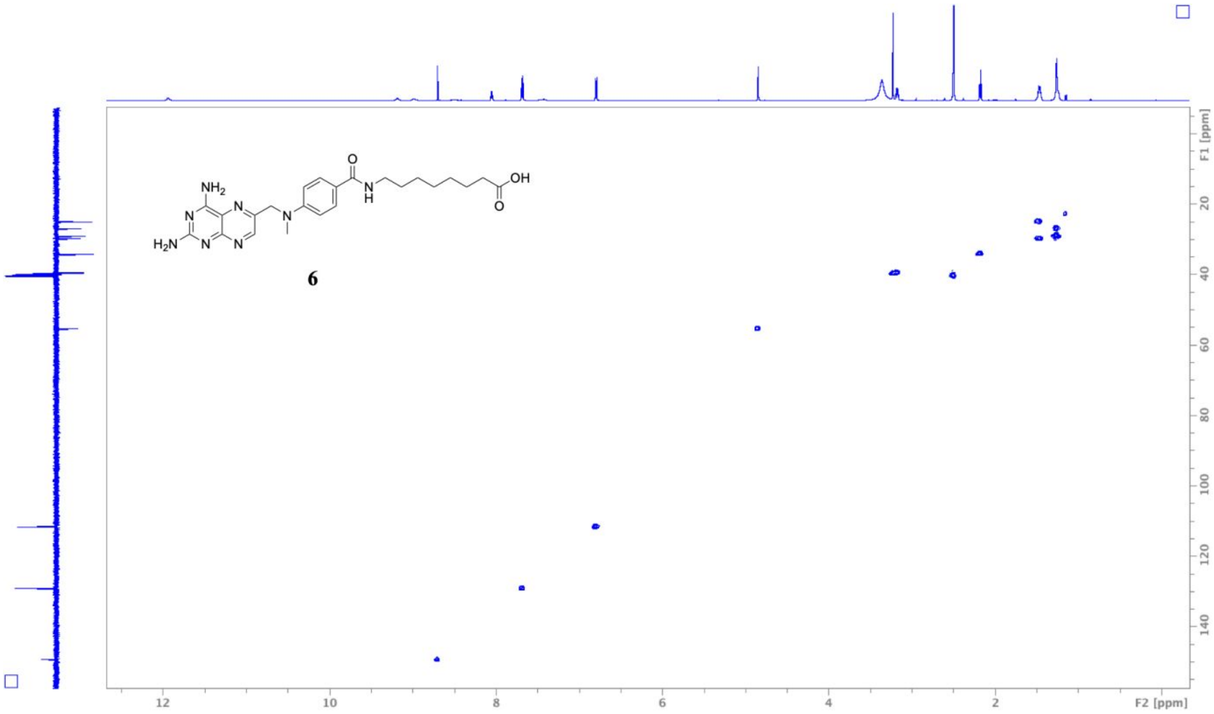

Figure S39. The ^{1}H-^{13}C HSQC NMR spectrum of **6** in DMSO-d_6

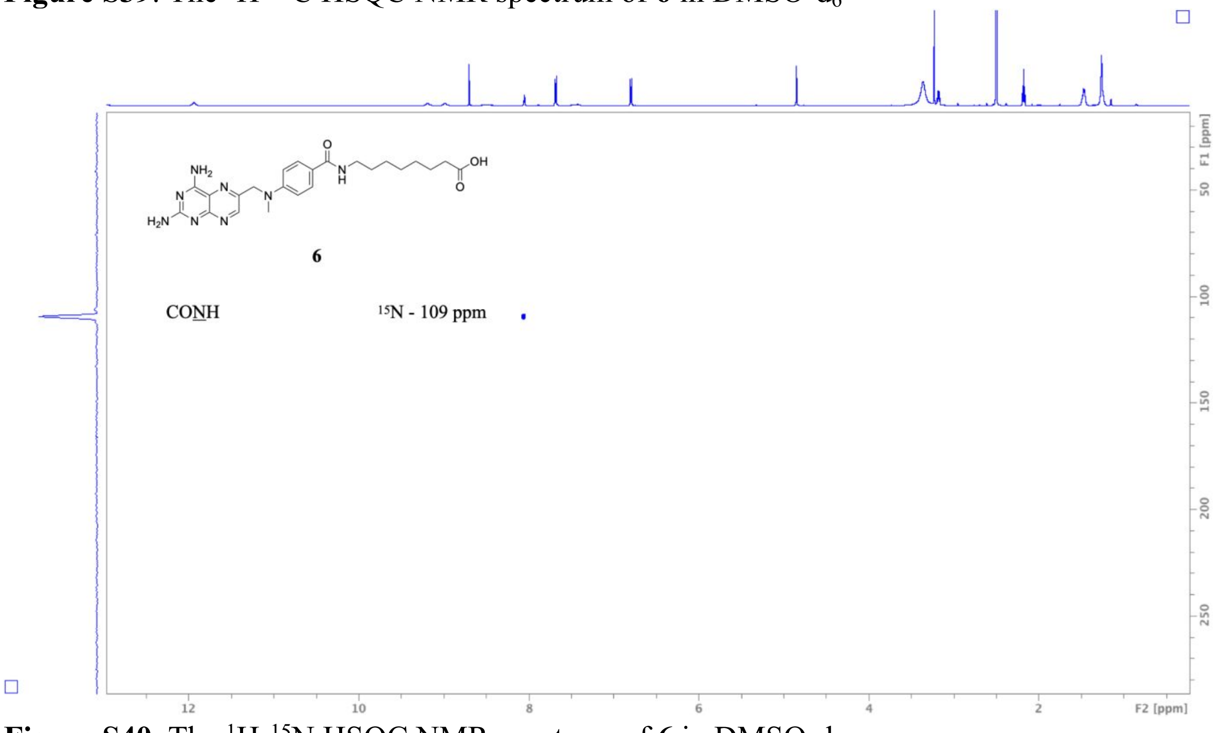

Figure S40. The ^{1}H-^{15}N HSQC NMR spectrum of **6** in DMSO-d_6

Table S1. Comparison of the RMSD values for heavy atoms between co-crystallized methotrexate and the docked molecule.

Pose	ASP	CHEMPLP	CS	GS
1	1.3225	0.6462	9.6534	0.7986
2	1.5184	1.1678	0.9600	0.7399
3	2.7863	0.6434	1.0309	0.9061
Mean	1.8757	0.8191	3.8814	0.8149

Table S2. Docking scores for compounds **1–7** in comparison to methotrexate.

Compound	ASP	CHEMPLP	CS	GS
methotrexate	61.7	98.8	24.7	95.3
**1**-P_R	64.0	106.4	31.7	92.1
**1**-P_S	49.9	90.3	30.3	88.9
**2**-P_R	69.2	111.4	23.3	97.0
**2**-P_S	66.2	92.8	18.9	94.9
**3**-P_R	55.9	107.2	9.9	85.8
**3**-P_S	58.6	108.3	10.8	92.6
**4**	61.2	86.7	26.0	76.4
**5**	60.6	88.4	26.9	79.1
**6**	62.5	90.3	26.6	87.9
**7**	62.2	92.2	27.2	84.9
